# Safety and Efficacy of Intraoperative Neuromonitoring: An Umbrella Review

**DOI:** 10.1002/hsr2.71370

**Published:** 2025-10-13

**Authors:** Aziz Rezapour, Naser Derakhshani, Seyedeh Narges Pouyan, Vahid Alipour, Seyed Jafar Ehsanzadeh, Arash Zare‐Sadeghi, Jalal Arabloo

**Affiliations:** ^1^ Health Management and Economics Research Center, Health Management Research Institute Iran University of Medical Sciences Tehran Iran; ^2^ Student Research Committee Tabriz University of Medical Sciences Tabriz Iran; ^3^ School of Health Management and Information Sciences Iran University of Medical Sciences Tehran Iran; ^4^ English Language Department, School of Health Management and Information Sciences Iran University of Medical Sciences Tehran Iran; ^5^ Medical Physics Department, School of Medicine Iran University of Medical Sciences Tehran Iran

**Keywords:** intraoperative neuromonitoring, intraoperative neurophysiological monitoring, IONM, motor‐evoked potentials, somatosensory‐evoked potentials

## Abstract

**Objective:**

Intraoperative neuromonitoring (IONM) plays a crucial role in several surgical procedures. This study aimed to review the safety and efficacy evidence of IONM technology.

**Methods:**

A comprehensive search was conducted in databases, including PubMed, Scopus, Web of Science core collection, Embase, and Cochrane library. Systematic reviews and meta‐analyses assessing clinical outcomes, safety, and diagnostic accuracy in patients undergoing neurosurgery with and without IONM were included. Methodological quality was assessed using the JBI tool. The present umbrella review followed the PRISMA guidelines.

**Results:**

A total of 48 systematic reviews and meta‐analyses were included in this study. The evidence demonstrated that IONM is clinically effective in reducing the risk of temporary recurrent laryngeal nerve paralysis and overall recurrent laryngeal nerve injury after thyroidectomy. Moreover, the use of IONM during aneurysm surgery was associated with fewer neurological complications. In carotid endarterectomy surgery, the combined assessment of electroencephalogram (EEG) and somatosensory‐evoked potentials (SSEP) as warning criteria exhibited higher sensitivity compared to EEG or SSEP alone. Notably, bispectral index (BIS)‐guided anesthesia did not show a reduction in consciousness risk. In procedures such as spinal, cervical, tumor resection, lumbar surgery, and deformity correction surgeries, IONM techniques demonstrated relative sensitivity in detecting surgery‐related nerve damage.

**Conclusion:**

Due to the heterogeneity in study types, variable warning thresholds, and limited strong clinical evidence, the efficacy of IONM remains uncertain and necessitates further investigation in this field. Future prospective studies and large‐scale randomized controlled trials are necessary to establish a definitive conclusion.

## 1 Introduction

Intraoperative neurophysiological monitoring (IONM) has become a crucial component in numerous surgical procedures since the commercial development of IONM devices in the early 1980s. Technological advancements in the past 15 years have led to the evolution of monitoring techniques, expanding its potential applications and increasing its popularity [1]. IONM involves the use of neurophysiological recording techniques to detect changes in the functional state of nerve tissues during surgery, with the underlying principle being that electrical activity changes occur before permanent nerve damage occurs [2].

Research has demonstrated that IONM provides important surgical support by identifying neural structures and reducing the risk of permanent nerve damage during surgery, thus improving patient safety. Various methods are employed in IONM, each with its own specific application [2, 3]. These techniques have been found to significantly reduce postoperative injuries without introducing additional risks, ultimately reducing healthcare costs. Evidence has shown that IONM utilizes a range of techniques, such as evoked motor potentials (MEPs), sensory evoked potentials (SSEPs), electroencephalography (EEG), electromyography (EMG), auditory evoked potentials (AEP), and vision evoked potentials (VEPs), to prevent and minimize damage to the patient's nervous system, while also aiding surgeons and anesthesiologist [1, 4, 5, 6, 7, 8]. IONM techniques are widely used in spinal surgery, certain brain surgeries, carotid endarterectomy, aortic aneurysms, ENT surgeries such as acoustic neuroma (vestibular schwannoma), parotidectomy, and nerve surgeries [9].

In recent years, several systematic reviews and meta‐analyses evaluating the safety and cost‐effectiveness of IONM have been published [10, 11, 12, 13, 14]. However, discrepancies in the conclusions of these studies have created confusion surrounding clinical decision‐making and the appropriate policies related to IONM. While systematic reviews and meta‐analyses are considered the best evidence for evaluating treatment effectiveness and developing clinical guidelines, only high‐quality systematic reviews should be utilized as the basis for making decisions. Unfortunately, there is currently no comprehensive overview of the effects of IONM in various surgeries, further emphasizing the need for further research.

A study by Ney et al. demonstrated that the use of IONM in spinal procedures reduces the relative risk of neurological complications by 49% and estimated the cost of preventing a nerve injury to be $63,387 [10]. In contrast, Traynelis et al. found that using IONM did not lead to significant cost savings exceeding $1 million [15]. Wang et al.'s economic evaluation study concluded that IONM is cost‐effective for preventing permanent recurrent laryngeal nerve (RLN) injuries [16], while Sanabria et al.'s study indicated that routine use of IONM in thyroidectomy with low risk of recurrent laryngeal nerve injury is not cost‐effective in the Colombian health system [17]. Given the discrepancies in results, potential biases in studies, and the increasing use of IONM as a potential standard of care, particularly in thyroid surgeries, there is a critical need for a systematic review evaluating the safety and effectiveness of this technology in various surgeries [13, 18]. Furthermore, evaluating the quality of studies is of utmost importance.

Therefore, the present study aims to review the safety and effectiveness evidence of intraoperative neuromonitoring technology across various surgical procedures, while also assessing the quality of the studies.

## 2 Methods

We conducted and reported an umbrella review on intraoperative neuromonitoring technology, focusing on its safety and clinical efficacy following the guidelines outlined in the book “A Systematic Review to Support Evidence‐Based Medicine” and the principles of the PRISMA statement [19, 20].

### 2.1 Information Sources

To collect the necessary data for this study, we conducted searches in various databases including PubMed, Scopus, Web of Science Core Collection, Embase, Cochrane Reviews, and Google Scholar without any time restrictions. We also employed specific search strategies for each database, utilizing a combination of relevant keywords and medical subject headings (MeSH). Additionally, we recorded all extracted studies in Endnote (version X8; Thomson Reuters). The final search for this study was performed on February 6, 2021. The search strategies for each database are presented in Table 1.

**Table 1:** Search strategy and summary of search results for each database and for systematic review and meta‐analysis studies.

Database	Search strategy	# Results
PubMed	“Intraoperative Neurophysiological Monitoring”[Mesh] OR “neurophysiologic* monitoring*”[tiab] OR “Intraoperative Neurophysiologic*”[tiab] OR neuromonitoring[tiab] Filters: Meta‐Analysis, Systematic Review	83
Embase	(‘neuromonitoring’/exp OR ‘intraoperative neurophysiological monitoring’/exp OR ‘intraoperative neurophysiological monitoring’ OR ‘intraoperative neuromonitoring’/exp OR ‘intraoperative neuromonitoring’ OR ‘neurophysiological monitoring’/exp OR ‘neurophysiological monitoring’) AND ‘human’/de AND (‘meta analysis’/de OR ‘meta analysis topic’/de OR ‘systematic review’/de OR ‘systematic review topic’/de)	257
Web of Science core collection	TS = (“Intraoperative Neurophysiologic* Monitoring” OR “neurophysiologic* monitoring*” OR “Intraoperative Neurophysiologic*” OR neuromonitoring) AND TS = (“meta analysis” OR “meta‐analysis” OR “systematic review”) Timespan: All years. Indexes: SCI‐EXPANDED, SSCI, A&HCI, ESCI.	85
Scopus	(TITLE‐ABS‐KEY ((“neuromonitoring” OR “intraoperative neurophysiological monitoring” OR “intraoperative neuromonitoring” OR “neurophysiological monitoring”)) AND TITLE‐ABS‐KEY (“meta analysis” OR “meta‐analysis” OR “systematic review”))	290
Cochrane Reviews	MeSH descriptor: [Intraoperative Neurophysiological Monitoring] explode all trees	1
Additional records identified through other sources		10
Total		726

### 2.2 Eligibility Criteria

Population: Patients undergoing spinal surgery, brain surgeries, carotid endarterectomy, aortic aneurysm repair, ENT surgeries (e.g., acoustic neuroma, parotidectomy), and neurosurgery.

Intervention and comparator: Studies comparing neuromonitoring technology with visual nerve identification or studies not utilizing the technology to diagnose and prevent nerve damage.

Type of outcomes: Studies reporting at least one or more of the following outcomes:

- Clinical effectiveness and safety.
- Reduction in the risk of injury and neurological complications during surgery.
- Diagnostic accuracy (sensitivity and specificity).

Type of studies: Systematic reviews and meta‐analyses.

Linguistic limitations: Only full‐text studies in English were included.

### 2.3 Exclusion Criteria

We excluded review studies, editorials, letters to editors, abstracts, unpublished gray texts such as dissertations and theses, and animal studies. Studies published in languages other than English were also excluded. Additionally, duplicate studies that reported the same results in multiple articles were excluded.

### 2.4 Review Process

After collecting the extracted articles and removing duplicates, we reviewed the titles of all articles and excluded those that did not align with the objectives of the study. Next, we screened the abstracts and full‐texts of the articles, excluding those that did not meet the inclusion criteria and had little relevance to the study objectives. The reasons for exclusion were documented. The entire systematic review process was conducted independently by two authors of the study, and any disagreements between them were resolved by involving a third party.

### 2.5 Quality Assessment

We utilized the JBI Critical Appraisal Checklist for Systematic Reviews and Research Syntheses to assess the quality of included systematic review and meta‐analysis studies [20]. This tool, which includes 11 questions and four answer options (YES, No, Unclear, Not applicable) for each question, was used to assess the risk of bias. Two authors of the study independently performed the quality assessment of the included studies, with any discrepancies being resolved by a third party.

### 2.6 Data Extraction

We entered the extracted data into a form and data table specifically developed for this study. To ensure the adequacy and effectiveness of this form, we piloted it using data from five articles. The form included the following information: authors' names, publication year, study type (systematic review/meta‐analysis), searched databases, time range of included studies, patient population, treatment indication, number and type of included studies, quality assessment, quality assessment tools, statistical models for meta‐analysis, and outcomes (e.g., risk/odds ratio). Two authors of the study independently performed data extraction for each included study, with supervision from a third party.

### 2.7 Data Analysis

Due to significant heterogeneity among the included studies in terms of design and statistical cut off, no data reanalysis (i.e., meta‐analysis) was conducted. Instead, we employed a narrative synthesis of the findings based on JBI recommendations for umbrella reviews [20]. The findings were organized in descriptive tables, which included key characteristics of the methodology and key findings.

## 3 Results

### 3.1 Overview of Included Studies

**Figure 1:**
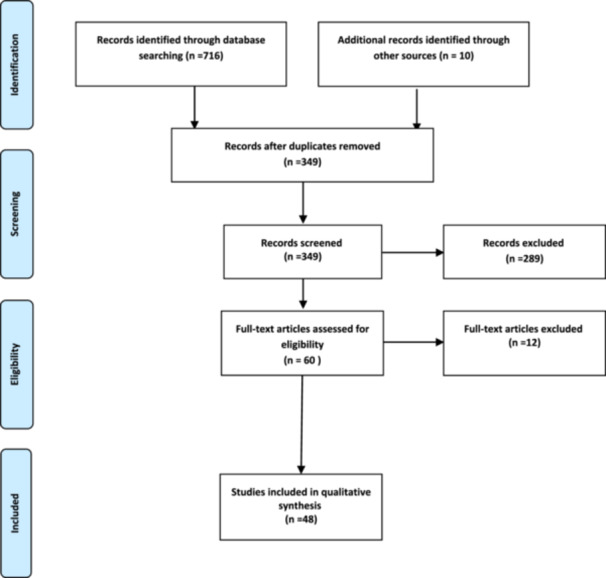
Flow diagram of the searches and inclusion process.

A total of 726 studies were extracted from databases and other sources. After removing duplicates, 349 articles remained. Subsequently, 60 studies were screened and 12 articles were excluded based on the inclusion/exclusion criteria. Ultimately, 48 studies were included in the analysis of this study (Figure 1).

**Figure 2:**
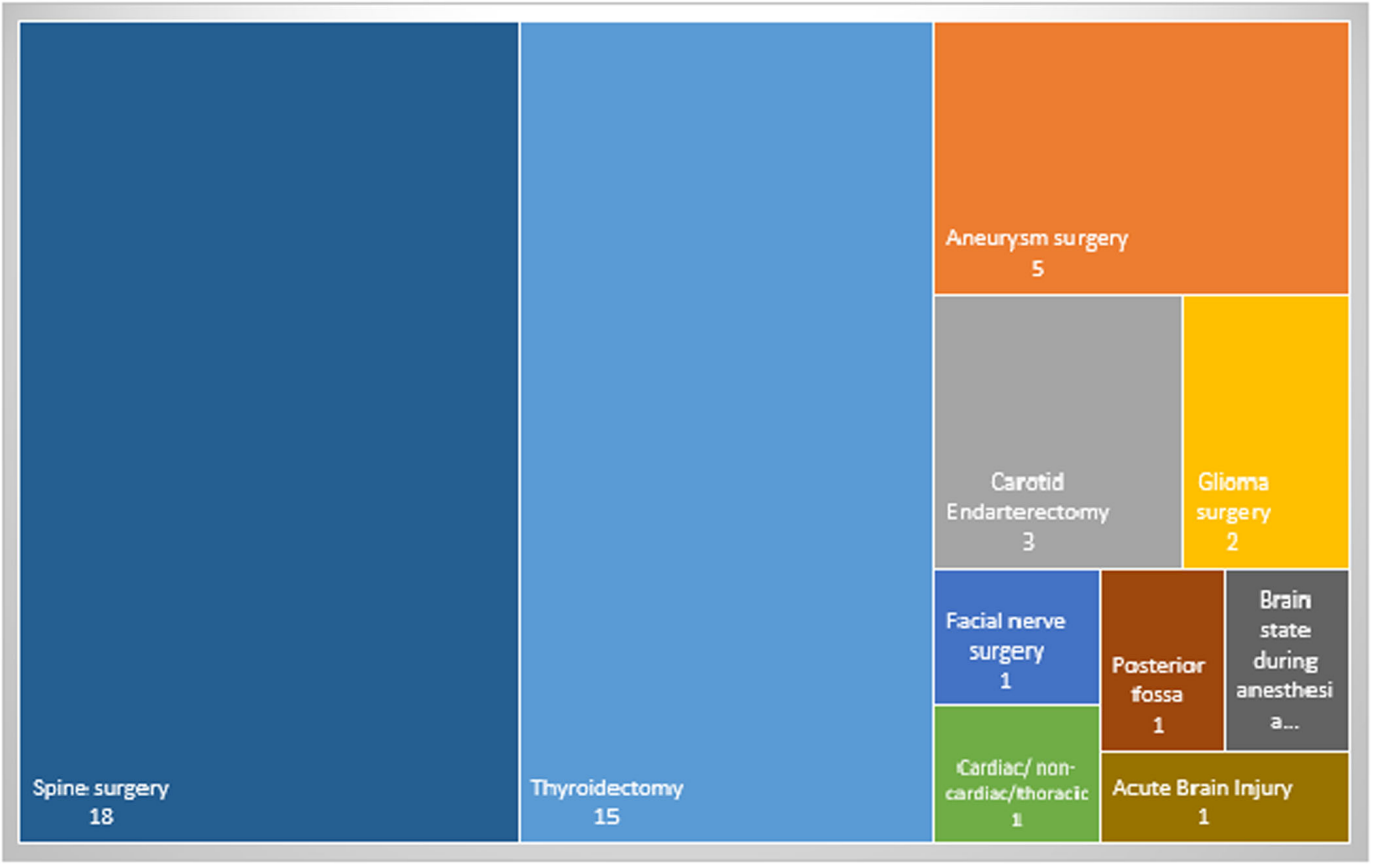
An overview of the included studies in terms of using IONM in various surgeries.

Figure 2 provides an overview of the included studies that used intraoperative neuromonitoring (IONM) in various surgeries. These surgeries include thyroidectomy (*n* = 15) [13, 14, 21, 22, 23, 24, 25, 26, 27, 28, 29, 30, 31, 32, 33], aneurysm surgery (*n* = 5) [9, 34, 35, 36, 37], carotid endarterectomy (*n* = 3) [38, 39, 40], glioma surgery (*n* = 2) [41, 42], facial nerve surgery (*n* = 1) [43], thoracic/cardiac/noncardiac surgeries (*n* = 1) [44], monitoring of brain status during anesthesia [45], acute brain injury (ABI) (*n* = 1) [46], foster posterior surgery (*n* = 1) [43] and spinal surgery (*n* = 18) [40, 47, 48, 49, 50, 51, 52, 53, 54, 55, 56, 57, 58, 59, 60, 61, 62, 63].

### 3.2 Quality Assessment of Studies

**Figure 3:**
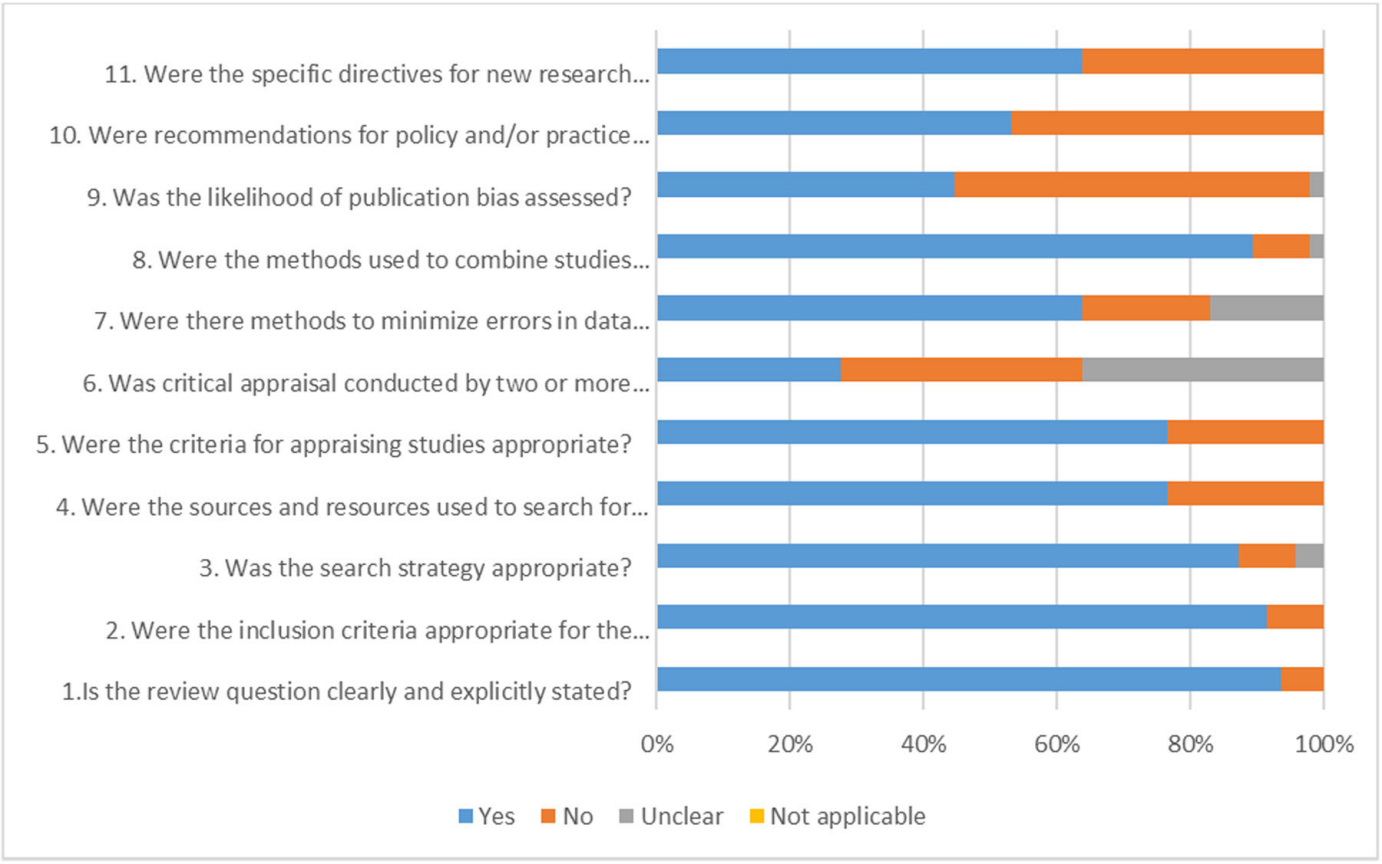
Results of quality assessment.

Figure 3 presents the results of the quality assessment of the included studies. The findings indicate that while the studies were properly designed and collected appropriate data, most of them were found to be weak in terms of three criteria: independent quality assessment conducted by multiple judges, evaluation of publication bias, and provision of policy and practical recommendations (Appendix 1).

### 3.3 Effectiveness of IONM in Different Surgeries

#### 3.3.1 Thyroid Surgery (Thyroidectomy)

Fifteen studies on thyroidectomy were published between 2011 and 2019 [13, 14, 21, 22, 23, 24, 25, 26, 27, 28, 29, 30, 31, 32, 33]. Among these studies, five reported statistically significant results in favor of using IONM for temporary recurrent laryngeal nerve paralysis [14, 25, 30, 31, 32], while only two studies demonstrated significant results for permanent paralysis [24, 30]. Other studies did not show statistically significant results for these indicators [13, 23, 26, 29, 33] (Table 2 and Appendix 2).

**Table 2:** Characteristics of studies on the use of IONM during thyroid surgery (thyroidectomy).

Author & year	Type of study	Study objective	Databases	Searching time	Number and type of included studies	Quality assessment	Quality assessment tool	Analysis	Type of statistical analysis	Measures
[21]	Systematic review and meta‐analysis	Evaluation of the effects of IONM compared with visual identification of the nerve to prevent RILN injury in adults undergoing thyroid surgery.	CENTRAL, MEDLINE, Embase, ICTRP Search Portal, and Clinicaltrials.gov. (21 August 2018)	2013–2018	5 Randomized controlled trial study (RCT)	Yes	GRADE	Yes	Random effect model	Risk ratio
[22]	Systematic review	Investigation and evaluation of the use of IONM in endoscopic thyroidectomy	CENTRAL, MEDLINE, Cochrane, and EMBASE (from January 1, 2000, to September 1, 2016)	2000–2015	9 studies (6 retrospective studies/3 randomized prospective studies)	No	No	No	No	—
[33]	Meta‐analysis	Comparison of the effect of the recurrent laryngeal nerve (RLN) compared to RLN identification alone on the actual degree of vocal cord paralysis after thyroidectomy.	MEDLINE (1966‐July 2008), EMBASE (1980‐July 2008), Cochrane Central Register of Clinical Trials (CENTRAL), Cochrane Database of Systematic Reviews, clinicaltrials.gov, and The National Guideline Clearinghouse databases	2009– 1991	43 studies (1 Randomized clinical trial، 7 comparative trials 34 case series)	Yes	CONSORT	Yes	Random effect model	Odd ratio
[23]	Meta‐analysis	Conducting a meta‐analysis of the combined results of individual studies to measure the frequency of RLN and EBSLN injuries in patients undergoing neuromonitoring during thyroidectomy compared to the usual method of identification	The Cochrane Central Register of Controlled Trials (CENTRAL) on The Cochrane Library (2012), The National Library of Medicine (PubMed) (1966–December 2012), EMBASE (1980–December 2012) and The Latin American and Caribbean Health Sciences Library (LILACS) (1980–December 2012).	2009–2012	6 Randomized controlled trial study	Yes	GRADE	Yes	Random effect model	Risk difference
[24]	Meta‐analysis	To evaluate whether IONM can reduce the incidence of RLN palsy to a greater extent than visual identification of RLN alone in re‐thyroid surgeries	PubMed, SCIE and Wan Fang databases/studies published up to 31 August 2016.	2004–2014	9 studies (2 study prospective cohort7 study retrospective cohort)	Yes	Newcastle‐Ottawa Scale (NOS)	Yes	Random effect model	Risk ratio
[32]	Systematic review and meta‐analysis	To evaluate the role of IONM in reducing RLN paralysis during high‐risk thyroidectomy and to identify which high‐risk subgroups will benefit the most.	Pubmed, Medline, Embase and Cochrane central register of clinical trials (CENTRAL) from 1st January 2000 to 30th June 2015	2004–2014	10 studies (2 study prospective comparative and 8 study retrospective)	Yes	Newcastle‐Ottawa Scale (NOS)	Yes	Fixed effect model	Odd ratio
[14]	Meta‐analysis	Using the meta‐analysis method to evaluate the role of IN in helping thyroid surgery	PubMed, Embase, and the Cochrane library from January 1, 2004 to July 30, 2016.	2004– 2016	24 studies (4 Randomized controlled trial studies & 20 study RCS)	Yes	Cochrane Collaboration tool	Yes	Random effect model	Odd ratio
[25]	Meta‐analysis	To evaluate the effect of IONM during thyroid surgery	The web‐based PubMed database (1950 through April 2011), Embase (1974 through April 2011), and the Cochrane Central Register of Controlled Trials (CENTRAL, The Cochrane Library, Issue 2 of 4, April 2011)	1992– 2009	14 (2 randomized clinical trials & 12 comparative studies)	Yes	Cochrane Risk of Bias tool	Yes	Random effect model	Odd ratio
[26]	Meta‐analysis	Determining the benefit of using intermittent neuromonitoring during surgery to prevent permanent nerve paralysis	PubMed, Scopus, and Cochrane Central Register of Controlled Trials until August 2014	2004–2014	14 studies (4 randomized controlled trials) & (10 studies on‐randomized controlled trials (NRS))	Yes	Cochrane Collaboration's tool	Yes	Fixed effect model	Risk ratio/risk difference
[27]	Systematic review and meta‐analysis	Evaluation of the advantage of IONM in identifying EBSLN during thyroid surgery	MEDLINE, PubMed, Web of Science, and Cochrane Library January 1, 1995, through July 1, 2018	2009–2016	7 study	Yes	Cochrane Collaboration tool	Yes	Random effect model	risk ratio
[28]	Systematic review and meta‐analysis	Risk estimation of bilateral RLN paralysis with and without intraoperative neuromonitoring	PubMed, Scopus (EMBASE), and the Cochrane Library in the period 2000‐2014	2000–2014	40 studies (…. clinical trials, …. cohort studies case series)	No	No	Yes	Random effect model	Adjusted incidence/risk ratio
[29]	Systematic review and meta‐analysis	Assessing the potential improvement of IONM versus visual identification of RLN alone (VA) in reducing the incidence of vocal cord paralysis	Embase, Medline, Cochrane, PubMed, and Google Scholar databases in August 2013	2004–2013	20 studies (3 prospective randomized trials,7 prospective trials & 10 studies observational retrospective)	Yes	MOOSE	Yes	Fixed effect model	Standardized Mean Difference
[13]	Systematic review	Evaluating the effectiveness of intraoperative neuromonitoring (IONM) in preventing recurrent laryngeal nerve palsy (RLNP) during thyroid surgery.	MEDLINE, EMBASE, and PubMed from 1999 forwards	2004–2014	17 studies (12 comparative studies ، 1 randomized clinical trial ، 2 non‐randomized clinical trials & 2 studies)	No	No	No	Not reported	Not reported
[30]	Meta‐analysis	Determination of the effects of IONM in thyroidectomy	MEDLINE (PubMed), BIOSIS Previews (ISI Web of Knowledge) and Cochrane library from January 1980 to July 2017	2002–2017	34 studies (3 randomized controlled trials 3 & non‐randomized trials)	Yes	Newcastle‐Ottawa Scale (NOS)	Yes	Random effect model	Risk ratio/risk difference
[31]	Meta‐analysis	Evaluation of the risk of temporary or permanent RLN damage in thyroid surgery with or without IONM	PubMed and Ovid, and the Cochrane Library database from January 1994 to February 2012	2002–2009	8 studies (2 randomized trials 6 comparative study‐randomized)	No	No	Yes	Fixed effect model	Risk ratio

#### 3.3.2 Cerebral Aneurysm Surgery, Thoracoabdominal, and Aortic Aneurysm Surgery (DTA and TAAAR)

Studies conducted between 2015 and 2020 indicated that using IONM in these surgeries influenced outcomes such as the incidence of new neurological defects, diagnosis, clinical attacks like delayed cerebral ischemia, mortality, morbidity, and prognosis of postoperative neurological defects [9, 34, 35, 36, 37]. However, there is no consensus on the use of motor evoked potentials (MEPs), and current evidence does not strongly support their use. Nonetheless, using MEPs during DTA and TAAAR is considered safe with minimal side effects [9] (Table 3 and Appendix 3).

**Table 3:** Characteristics of studies on the use of IONM during cerebral aneurysm surgery.

Author & Year	Type of study	Study objective	Databases	Searching time	Number and type of included studies	Quality assessment	Quality assessment tool	Analysis	Type of statistical analysis	Index
[34]	Systematic review و meta‐analysis	To evaluate whether IOM can prevent nerve damage during clipping of intracranial aneurysm	PubMed/Medline, Scopus, and Cochrane databases. January 1999 and January 2019	2010–2019	4 studies (three studies including group retrospective studies (before and after using IOM) and one prospective cohort study to evaluate IOM changes during MCA aneurysm surgery	Yes	MINORS	Yes	Random effect model	Odd ratio
[35]	Systematic review	Evaluation: (a) diagnostic accuracy of cEEG as a confirmatory test, (b) prognostic value of EEG patterns indicative of seizures and DCI, and (c) efficacy of neuromonitoring using cEEG in terms of improving clinical outcome following SAH	Cochrane Central Register of Controlled Trials (The Cochrane Library), Medline (PubMed), EMBASE, Scopus, from January 1, 1980 to June 15,2014	1991–2014	18 studies (single‐center case series (including a randomized clinical trial)	Yes	QUADAS‐2	No	—	
[36]	Systematic review و meta‐analysis	Investigating the diagnostic accuracy of different evoked potential monitoring techniques in predicting postoperative neurological deficits in brain aneurysm surgery	MEDLINE, Embase and Cochrane databases 1983 through March 2016	1994–2013	15 prospective study	Yes	QUADAS	Yes	Random effect model	
[37]	Meta‐analysis	To assess the accuracy of the intraoperative evoked potential (EP) diagnostic test (DTA) for the diagnosis of brain injury during brain aneurysm surgery.	EDLINE, EMBASE, LILACS, IndMed and a variety of other sources from 1 January 1960 to 5 January 2016 (last updated on 27 June 2018)	1987– 2017	35 observational studies (prospective and retrospective clinical studies)	Yes	QUADAS‐2	Yes	Random effect model	
[9]	Systematic review	Evaluation of the benefit and application of neuromonitoring in descending aneurysm repair surgery and thoracoedema (DTA and TAAAR).	OVID Medline, PUBMED, Scopus, and COCHRANE (from their dates of inception until February 2014)	1999– 2013	15 prospective and retrospective study	No	—	No	—	—

#### 3.3.3 Carotid Endarterectomy

Results from studies conducted between 2015 and 2018 revealed that the outcomes of using IONM in carotid endarterectomy include sensitivity, diagnostic risk ratio [39, 64], stroke risk prediction [38], and the potential of changes in somatosensory evoked potentials (SSEP) to predict stroke in the 30‐day postoperative period [38]. Multimodal monitoring with changes in electroencephalography (EEG) or SSEP as warning criteria showed higher sensitivity compared to EEG or SSEP alone [39]. EEG monitoring was found to be a strong predictor of strokes in carotid endarterectomy (Table 4).

**Table 4:** Characteristics of studies using IONM during carotid endarterectomy, glioma surgery, cardiac/noncardiac/thoracic surgery, cranial base tumor surgery, posterior fossa surgery, acute brain injury, and brain condition during anesthesia.

Author & year	Type of surgery	Type of study	Study objective	Databases	Searching time	Number and type of included studies	Quality assessment	Quality assessment tool	Analysis	Type of statistical analysis
[38]	Carotid endarterectomy	Meta‐analysis	Evaluating the efficacy of intraoperative SSEP change in predicting the risk of stroke in the postoperative period: more than 24 h but within 30 days	PubMed, Web of Science, and Embase	1985–2015	25 prospective and retrospective studies	No	No	Yes	Bivariate normal model
[39]	Carotid endarterectomy	Meta‐analysis	To determine whether multimodal monitoring leads to increased diagnostic sensitivity and accuracy?	Embase, PubMed, and Web of Science databases from 1945 through 26th March 2015	1995–2012	4 prospective and retrospective studies	Yes	QUADAS 2	Yes	Bivariate model
[50]	Carotid endarterectomy	Meta‐analysis	To determine the diagnostic accuracy of electroencephalogram (EEG) in predicting postoperative strokes through a meta‐analysis of existing literature.	PubMed and Web of Science databases for relevant literature from 1945 through 8 August 2014	1975– 2007	30 prospective and retrospective clinical trial studies	Yes	QUADAS	Yes	Bivariate model
[41]	Glioma surgery	Systematic review & meta‐analysis	Investigation of early and permanent postoperative defects in patients who underwent insular glioma surgery using awake craniotomy with direct electrical stimulation (DES) versus surgery under general anesthesia.	PubMed, Ovid MEDLINE, and Ovid EMBASE (January 1990 to January 2018)	1997–2016	5 prospective and 3 retrospective studies	Yes	Newcastle‐Ottawa Scale	Yes	Random effect model
[42]	Glioma surgery	Systematic review	Investigating the effect of assistive technologies during surgery on the rate of risk (EOR) in glioma surgery, compared to conventional unassisted surgery.	MEDLINE (PubMed), Scopus, Web of Science, and SciELO (2006 ‐2014)	2006– 2013	6 prospective controlled studies	No	No	No	No
[44]	Cardiac/noncardiac/chest surgery	Systematic review & meta‐analysis	Evaluating the effectiveness of brain monitoring of the depth of anesthesia in reducing postoperative cognitive function and postoperative delirium	MEDLINE, EMBASE, and Cochrane Library databases NA	2011–2013	5 clinical trials	No	No	Yes	Random effect model
[43]	Tumor surgery of skull base and Cerebellopontine Angle	Systematic review	To review the current literature with emphasis on all aspects of FN monitoring for Cerebellopontine Angle and skull base tumor from description to NO success in predicting the performance of standard and emerging monitoring methods.	PubMed (up to February 2011)	DES: (1979 –2010) free‐running EMG: (1987‐2010) FMEP: (2001–2011)	In DES, 27 retrospective studies and 35 prospective studies/in EMG 6 retrospective studies and seven prospective studies/in FMEP 3 retrospective studies and 3 prospective studies	No	No	No	No
[65]	Posterior fossa surgery	Systematic review	The review of the available evidence has been carried out with an objective study; an intraoperative examination of the neural circuits involved in the pathophysiology of cerebellar mutism syndrome	PubMed central (Searching Date Not Mentioned)	not mentioned	2 studies (type of study not mentioned)	Not mentioned	Not mentioned	Not mentioned	Not mentioned
[46]	Acute brain injury	Systematic review	To determine the optimal use and indications of electroencephalography (EEG) in the intensive care management of acute brain injury (ABI).	PubMed (January 1990 through August 15, 2013)	1979‐2013	3 clinical trial studies, 1 case study, 80 prospective observational studies, 78 retrospective studies	Yes	GRADE	No	No
[45]	The state of the brain during anesthesia	Systematic review & meta‐analysis	Will anesthesia management by processed EEG reduce the rate of unwanted consciousness with recall during anesthesia, postoperative delirium, postoperative neurocognitive disorders, and ultimately, long‐term mortality after surgery, or No.	Ovid MEDLINE/EMBASE/Cochrane Central Register of Controlled Trials/Cochrane Database of Systematic Reviews/PubMed/Web of Science (2000 to October 1, 2018)	2003‐2019	15 clinical trials	Yes	GRADE	Yes	Random effect model

#### 3.3.4 Glioma Surgery

Two studies conducted in 2015 and 2020 on glioma surgery assessed clinical outcomes such as postoperative complications, functional status, survival without disease progression, and extent of resection (EOR) [41, 42]. One study reported that the rate of primary postoperative failure after awake surgery with DES was higher than general anesthesia. However, the rate of persistent postoperative failure was lower in the awake DES subgroup. Also, no significant relationship was observed among the rate of postoperative failure, NIOM, intraoperative navigation, year of the study, and tumor histology [41].

Observational studies evaluating IONM in glioma surgery have shown an improvement in EOR for both low‐ and high‐grade glioma (Table 5). Results of the only controlled prospective study showed that in IONM‐assisted surgery, the level of EOR for tumors in eloquent areas was not significantly different from the EOR for tumors in non‐eloquent areas. Overall, results confirmed that there was no reliable evidence regarding the use of IONM in glioma surgery [42].

**Table 5:** Characteristics of the studies included in the use of IONM during spinal surgeries.

Author & year	Type of surgery	Type of study	Study objective	Databases	Searching time	Number and type of included studies	Quality assessment	Quality assessment tool	Analysis	The statistical model used in analysis
[66]	Spine deformity surgery	Structural causality model and meta‐analysis	Improving MEP performance in deformity surgeries considering potential confounders	Embase from inception to January 2019	2002–2019	21 cohort studies	No	—	Yes	Structural causal model (SCM)
[56]	Pedicle screw placement	Systematic review & meta‐analysis	To determine the ability and reliability of tEMG technology to identify malpositioned pedicle screws	the US National Library of Medicine, the Web of Science Core Collection database, and the Cochrane Central Register of Controlled Trials for PS studies	1994–2014	26 studies	Yes	the Downs and Black checklist	Yes	In the performed meta‐analysis, sensitivity, specificity, overall, and subgroup ROC AUC were calculated. Publication bias was also assessed through 1‐tailed funnel plots
[47]	Spinal cord tumor surgery in Iran modular extra modular	Single‐center retrospective cohort and meta‐analysis	Summary of clinical outcomes of IONM in patients with ID‐EM spinal tumors	PubMed, Embase, Web of Science, and Scopus databases January 1980, to September 6, 2018	2015–2010	5 Retrospective	No	Duval and Tweedie's trim and fill test (publication bias)	Yes	Pooled diagnostic accuracy was assessed with a 95% confidence interval. A random effect model was used to estimate the cumulative diagnostic accuracy. Publication bias was assessed by drawing funnel plots, where the x‐ and y‐axes were the logit of event rates and standard errors of each study, respectively, and Duval and Tweedie's trim and fill test to determine the number of possible missing articles and the diagnostic value of the adjustment. has been used
[55]	Cervical decompression surgery	Systematic review	Reviewing the evidence related to the use of technology in cervical decompression surgery in the degenerative environment and trying to identify the best‐supported applications	PubMed and MEDLINE databases and Cochrane Central Registry of Controlled Trials (from March to July 2017)	2017–2004	8 [randomized controlled trials (RCTs), case series (CS), retrospective case series (RCS), and prospective cohort studies (PCS)]	Yes	American Academy of Orthopedic Surgeons (AAOS)	No	No
[63]	Spine surgery	Systematic review & meta‐analysis	Evaluation of IONM in the prevention of spinal cord injuries	MEDLINE (PubMed), Embase, Lilacs, and Cochrane Central Register databases of randomized assays (January 2007 to September 2017)	2016–2006	6 comparative clinical studies	Yes	MINORS	یله	Random effect model
[58]	Carino‐encephalic surgery, spine surgery, peripheral vascular surgery	Systematic review	Providing some recommendations based on the best available evidence, with study objective to standardize methods to support IONM in reducing the risk of secondary nerve damage in patients undergoing Carino‐encephalic surgery, spine surgery, peripheral vascular surgery	American University of Beirut Medical Center (one document), PubMed Central (four documents), Springer Link (eight documents), OVIDSP (seven documents), Researchgate (two documents), Science Direct (11 documents) and Wiley Online Library (three documents) and Scottish Intercollegiate Guidelines Network (SIGN) systems to establish the level of evidence (LoE) and grade of recommendation (GoR).	2017–2009	1 guideline, 2 systematic review studies, 8 RCT studies, 44 observational studies, and 1 study without specific classification.	Yes	the CEPD and SIGN scales	No	—
[48]	Surgery of intramedullary spinal tumors	Systematic review & meta‐analysis	Summary and review of reported evidence on the use of IONM of spinal cord tumors	Embase, Medline Epub, Cochrane Central, Web of Science, and Google Scholar from January 2000 to February 2018	2018–2005	31 studies in qualitative analysis meta‐analysis of 15 studies	Yes	QUADAS II	Yes	Bivariate model
[50]	Anterior cervical procedures for spondylotic myelopathy	Meta‐analysis	Evaluation of different IONM techniques in anterior cervical procedures	MEDLINE and the Web of Science for studies published up to February 2013	2012–1994	22 random, cohort and observational studies	No		Yes	
[40]	Corrective surgeries for idiopathic scoliosis patients	Meta‐analysis	Evaluation of common warning criteria and diagnostic value of MEP changes in spine surgery	MEDLINE/PubMed database to determine eligible studies published before October 2014	2012–1998	12 retrospective cohort study	Yes	QUADAS‐2	Yes	Bivariate model
[40]	Spine surgery	Meta‐analysis	Evaluation of efficacy (TcMEP) in predicting impending nerve amputation during corrective spine surgery for patients with idiopathic scoliosis (IS)	PubMed/MEDLINE, Web of Science, and EMBASE from 1945 to January 2014	2014–1996	25 retrospective cohort study	Yes	QUADAS‐2	Yes	Univariate random effects comparison model
[50]	Deformity surgery for idiopathic scoliosis	Meta‐analysis	Objectives to determine the sensitivity, specificity, diagnostic Odd ratio, and area under the receiver operating characteristic (ROC) curve of intraoperative SSEP/TcMEP combinations in relation to neurological outcome in patients undergoing idiopathic scoliosis correction surgery.	Pubmed/MEDLINE, Web of Science, and Embase electronic databases from January 1974 through January 2015	2015–2007	7 studies	Yes	QUADAS‐2	Yes	Univariate random effects
[50]	Deformity surgery for idiopathic scoliosis	Meta‐analysis	Determination of diagnostic accuracy (SSEP) to predict postoperative neurological outcome in patients undergoing spinal deformity surgery for correction of adolescent idiopathic scoliosis (AIS).	MEDLINE and World Science databases from January 1950 through January 2014	2014–1983	15 retrospective cohort and prospective studies	Yes	QUADAS 2	Yes	Bivariate model
[57]	Cervical surgery	Systematic review & meta‐analysis	Evaluation of the sensitivity and specificity of neuromonitoring and the risk of neurological damage after anterior cervical spine surgery (ACSS) with and without (ION)	Medline, Embase, Cochrane Reviews, SCOPUS, Web‐of‐Science (Searching Date Not Mentioned)	2014–1996	9 retrospective studies including 26,357 patients	Yes	MINORS	Yes	Comparison of random effects/mixed effect logistic regression to compare the sensitivity and specificity of non‐modal and multimodal ION
[59]	Spine surgery	Systematic review	Validation review of recommended warning criteria for IONM	MEDLINE, Excerpta Medica data BASE (EMBASE), Cochrane Controlled Trials Registry, Google Scholar Database of Abstracts of Reviews of Effects (DARE), and Cumulative Index to Nursing and Allied Health Literature (CINAHL) databases after 1980.		52 cohort studies	No	—		—
[60]	Spinal nerve surgery and monitoring	Systematic review	Investigating the evidence that IONM in the subspinal nerve affects the prevalence of postoperative shoulder diseases and the prediction of functional outcomes.	Medline, Scopus and Cochrane databases	2012–1995	3 studies	Yes	—	No	—
[49]	Intramedullary spinal tumor surgery	Systematic review & meta‐analysis	Evaluation of the diagnostic value of IONM in identifying postoperative injuries in IMSCT	PubMed, MEDLINE December 31, 2015 and June 30, 2016)	2016–1993	15 retrospective cohort studies, 4 prospective cohort studies, and two case‐control studies	Yes	American Academy of Neurology Evidence Classification System	Yes	Bivariate model and random effects
[53]	Cervical surgery	Systematic review	Reviewing the evidence of the use of norm‐monitoring technology as a diagnostic tool to evaluate nerve function during surgery for cervical spondylotic myelopathy.	National Library of Medicine (from 1996 through 2005)		Not specified	No	No	No	No
[54]	Lumbar cervical, and thoracic surgery	Systematic review	Determining the sensitivity and characteristics of neuromonitoring to identify neurological injuries during spine surgery and evaluating the ability of this technique to improve clinical outcomes for patients during procedures.	MEDLINE, EMBASE, and Cochrane Collaborative Library (between 1990 and March 2009)	2009–1986	32 retrospective cohort studies	Yes	Grading of Recommendations Assessment, Development, and Evaluation (GRADE) criteria	No	No

#### 3.3.5 Facial Nerve (FN) Surgery

There is limited evidence regarding the use of IONM in FN surgery. While IONM may improve surgical outcomes by reducing morbidity, there is a lack of randomized controlled trials in this area. The lack of standardization in electrode assembly and excitation parameters prevents definitive conclusions about the best method. Additionally, studies comparing different criteria or multimodal monitoring and their impact on FN anatomical and functional preservation are lacking [43] (Appendixes 4, 5, and Table 2).

#### 3.3.6 Chest/Cardiac/Noncardiac Surgeries

Studies in this area focused on outcomes such as unwanted alertness during anesthesia, postoperative delirium, postoperative neurological disorders, and long‐term mortality. Anesthesia with bispectral index (BIS) or AEP was associated with a significant reduction in the risk of postoperative delirium and long‐term cognitive decline [44].

#### 3.3.7 Monitoring Brain Status During Anesthesia, Acute Brain Injury (ABI)

IONM in these surgeries aims to manage anesthesia using processed EEG and its impact on unwanted consciousness, postoperative delirium, postoperative neurological disorders, and long‐term mortality. Results showed that BIS‐based anesthesia reduced the risk of consciousness when intravenous anesthesia was used. The depth of anesthesia based on EEG monitoring had no effect on long‐term mortality. EEG should be considered in patients with ABI and unexplained persistent altered consciousness or coma in the ICU without acute cerebral complications [45].

#### 3.3.8 Posterior Fossa Surgery

The focus of studies in this area was on the neural circuits involved in Cerebellar mutism syndrome. Comparison of preoperative and postoperative transcranial magnetic stimulation of the cerebellum may predict the onset of CM syndrome. However, there is limited data on neurophysiology during cerebellar surgery, presenting an opportunity for further research and hypothesis testing using IONM [65] (Appendixes 4, 5, and Table 6).

**Table 6:** Summary of the findings of studies related to the use of IONM in the surgery of spinal tumors.

Author	Included studies for meta‐analysis	Overall pooled sample size	INOM modalities	Outcome measurement	The number and type of studies included	Sensitivity	Specificity	Other measures
[47]	5	103	SSEPs, MEPs, NMJB, free‐running EMG, and EEG	Sensitivity specificity Positive Predictive Value Negative Predictive Value	4 + 1 single center cohort	IONM overall pooled diagnostic value: 77.9% (95% CI 62.1%–88.3%),	IONM overall pooled diagnostic value :91.1% (95% CI 82.2%–95.8%),	Positive Predictive Value: 56.7% (95% CI 27.7%–81.7%), Negative Predictive Value: 95.7% (95% CI 88.5%–98.5%)
[48]	15	37	IONM (SEP, MEP, and mIONM. SEP)	Sensitivity specificity negative Likelihood Ratio Positive Likelihood Ratio Diagnostic Odds Ratio	31 qualitative analyses 15 studies in quantitative analysis	MEPs 0.838 [95% CI, 0.703–0.919] SSEP 0.808 [95% CI, 0.679–0.893] MIONM 0.835 [95% CI, 0.695–0.919]	MEPs: 0.829 [95% CI, 0.536–0.843] SSEP: 0.714 [95% CI, 0.668–0.921] MIONM: 0579 [95% CI, 0.441–0.736]	**Positive Likelihood Ratio** MEPs: 4.901 SSEP:2.825 MIONM:2.072 **negative Likelihood Ratio** MEPs: 0.195 SSEP:0.269 MIONM:0.276 **Diagnostic Odds Ratio** MEPs: 29.717 SSEP:12.077 MIONM:7.507
[49]	17	806	MEPs, SSEPs, dorsal column mapping, d‐waves, EMG	Sensitivity pooled sensitivity specificity pooled specificity pooled DOR	21 qualitative study analysis/17 meta‐analysis study 15 retrospective cohort studies, 4 prospective cohort studies, and two case‐control studies	MEPs: individual studies range 75% to 99%, pooled: 90% [95% CI, 84%–94%] SSEP individual studies range:70% to 95% Pooled: of 85% (95% CI, 75–91)	MEPs individual studies range: 27% to 97% pooled: 82% (95% CI, 70%–90%) SSEP: individual studies range:61% to 96% Pooled: 61% to 96%	Pooled DOR MEPS 55.7 (95% CI, 26.3–119 Pooled DOR SSEP 14.3 (95% CI, 5.47–37.3) MEPS the pooled area under the hsROC curve MEP:91.8% SSEP: 86.3%

#### 3.3.9 Spinal Surgery

Studies conducted between 2006 and 2020 in the field of spinal surgery examined the diagnostic value and therapeutic neuromonitoring techniques. Diagnostic evaluations focused on indicators such as sensitivity, specificity, true positive, false positive, true negative, false negative, and diagnostic odds ratio. Therapeutic evaluations assessed the risk of surgery‐related neurological damage and impairment, using indicators such as incidence rate and odds ratio. The majority of studies investigated deformity correction [40, 50, 51, 56, 66], removal of spinal cord tumors [47, 48, 49] and cervical surgery [53, 54, 55, 57, 59, 62, 63]. Also, some studies have extended the scope of review and meta‐analysis to spinal surgery in general [54, 58, 59, 60, 61, 63]. An appropriate warning criterion is challenging to define [61], and the clinical value of neuromonitoring remains uncertain [59] (Appendixes 6, 7, and Table 7).

**Table 7:** Summary of the findings of studies related to the use of IONM in spinal deformity correction surgeries.

Author	Publication year	Included studies for meta‐analysis	Overall pooled sample size	INOM modalities	Outcome measurement	The number and Type of studies included	Sensitivity	Specificity	Other measures
Thirumala et al.	2017	12	2102 patients with idiopathic scoliosis	TcMEP	Incidence of neurological deficits, sensitivity, specificity	Prospective, or retrospective cohort reviews	91% [95% CI 34%–100%	96% [95% CI 92%–98%	Diagnostic odds ratio:250 [95% CI 11–5767] AUV: 0.98.
Thirumala et al.	2016	7	2,052 patients with idiopathic scoliosis	SSEP and TcMEP	Sensitivity, specificity, and DOR		Pooled: 82.6% (95% CI: 56.7%–94.5%),	Pooled: 94.4% (95% CI: 85.1%–98.0%),	DOR: 106.16 (95% CI: 24.952 – 451.667), AUC: 0.928, diagnostic odds ratio
Thirumala et al.	2016	15	4763 procedures on idiopathic patients	SSEP	Sensitivity and specificity of somatosensory evoked potentials to predict neurological deficits	Prospective, or retrospective cohort reviews	84%, [95% CI 59%–95%]	Pooled:98%, [95%CI 97%–99%]	Diagnostic odds ratio: 340 (95% Cl 125–926) AUC: 0.0.99
Holdefer, R. N.	2020	21	5055 spine deformity surgeries.	MEPs, or MEPs and SEPs	Probability of a MEP deterioration which recovered by the end of the surgery, P(RSC), and the conditional probability of no new postoperative deficit given an RSC, P(NND | RSC), stratified by category of an intraoperative adverse event associated with the MEP deterioration	Cohort	—	—	Probability of no new motor deficit, P(NND): (*r* = 0.71, *p* < 0.001) P(RSC) for an alert associated with correction: 0.76 for osteotomies (0.48, *p* = 0.0008) for hypotension (0.92, *p* = 0.06) P(NND | RSC) for correction: 0.94 for positioning:(0.82) for osteotomies: (0.86) for hypotension:(1.0) odds predictor of no new motor deficits 25.2, *p* < 0.001

## 4 Discussion

The objective of this study was to assess the safety and clinical efficacy of intraoperative neuromonitoring (IONM) in various surgical procedures. Over the years, the use of neuromonitoring technology to prevent and minimize surgery‐related injuries and neurological complications has increased. This study reviewed 15 systematic review and meta‐analysis studies on the application of IONM in thyroidectomy. However, due to the heterogeneity among these studies, there were inconsistencies in the results.

Overall, the results of these studies showed that IONM was clinically effective in reducing the risk of temporary RLN paralysis and the overall risk of RLN paralysis after thyroidectomy. However, there was no advantage in reducing the risk of definitive RLN paralysis after thyroidectomy. The findings of this study also indicated that IONM had advantages in reoperations and high‐risk patients. It facilitated the diagnosis of RLN and External Branch of the Superior Laryngeal Nerve (EBSLN), confirmed their functional integrity, identified the site of nerve damage, and provided functional feedback postoperatively. IONM was also successful in reducing bilateral laryngeal paralysis by improving immunity in patients undergoing surgery [67].

Additionally, the results of studies demonstrated that IONM was a reliable tool for anatomical identification and functional monitoring of laryngeal nerve branches. It also had psychological effects on patients, increasing satisfaction and trust in physicians, which in turn affected the success of the surgery. However, one major challenge of using IONM was the need for significant training. Conventional laryngeal nerve detection techniques should not be abandoned, as IONM may not always be available and its cost requires careful evaluation [68]. Further prospective randomized studies are needed to confidently generalize the effectiveness and cost‐effectiveness of IONM.

The results of five systematic review and meta‐analysis studies on the use of IONM in cerebral aneurysm surgery showed that it led to fewer neurological defects during surgery, but there was no statistically significant difference in long‐term follow‐up between the neuromonitoring and control groups. While there is no consensus on the usefulness of IONM in cerebral aneurysms, the studies confirmed that neuromonitoring during motor evoked potentials (MEPs) was a safe procedure with few side effects.

Another important factor in using IONM in patients who have undergone carotid endarterectomy is that they are more likely to have a stroke within 24 h before and 30 days after surgery due to SSEP changes. Also, results showed that an increase in the predicted risk of stroke was associated with drastic changes in SSEP [38, 41]. Therefore, the absence of SSEP compared to changes in SSEP will lead to a higher risk of stroke after surgery; and in general, continuous changes in SSEP than transient changes in SSEP indicates a higher risk of stroke after surgery. However, these findings were more significant when the analyses were considered for two periods of 24 h after and before the surgery. This may also indicate the need for varying degrees of perfusion. However, these results have been expressed while heterogeneity has been high in the studies (*I* ^2^ > 20%) [39]. Reasons for heterogeneity in these studies included nonuniform reporting of outcome data, differences in follow‐up periods, and differences in area details and severity of stroke due to different protocols and standards. Therefore, the interpretation and generalization of the results of these studies should be taken cautiously [38]. Also, in terms of sensitivity of IONM in carotid endarterectomy surgery, simultaneous changes of both EEG or SSEP methods as warning criteria were more sensitive than EEG and SSEP alone. EEG monitoring has a high sensitivity in predicting stroke during surgery (perioperative stroke). Despite the qualitative evaluation of studies using the QUADAS‐2 tool, there have been limitations such as diffusion bias, differences in study design, bias in data collection and interpretation, and methodological differences between the studies [39, 64].

There is currently no reliable evidence on the use of IONM in glioma surgery. In a meta‐analysis study conducted in 2020, there was no significant relationship between the rate of postoperative failure and the amount of intraoperative neurophysiological monitoring. Also, in IONM‐assisted surgeries, the level of EOR for tumors in eloquent areas was not significantly different from EOR for tumors in non‐eloquent areas [41, 42]. In facial nerve surgery, intraoperative neuromonitoring may improve surgical outcomes, but there is a lack of randomized controlled trials and studies comparing different monitoring criteria [43].

In cardiac/noncardiac/thoracic surgeries, BIS or AEP anesthesia was found to reduce the risk of postoperative delirium and long‐term cognitive decline. To reduce the risk of POD, there is no difference between the ability of anesthesia with BIS and AEP. In addition, there was significant heterogeneity between studies in patients undergoing heart and chest surgery; however, no heterogeneity was observed in patients undergoing noncardiac surgery [44]. On the management of anesthesia by processed EEG and its effect on the level of unwanted consciousness with reminders during anesthesia, postoperative delirium, postoperative neurological disorders, and long‐term postoperative mortality, findings of reviewed studies showed that BIS‐based anesthesia does not reduce the risk of consciousness; however, BIS‐based anesthesia significantly reduces the risk of consciousness in patients receiving intravenous anesthesia. EEG or BIS monitoring reduced the risk of delirium, but there was significant heterogeneity (*I* ^2^ = 70.8%). Also, there was a significant increase in the risk of postoperative neurological disorders with BIS monitoring. Deep and light anesthesia based on EEG monitoring had no effect on long‐term mortality; also, significant heterogeneity was observed in studies [45]. Regarding the role of IONM in ABI, results showed that EEG should be unexplained and persistent in all patients with ABI and altered consciousness, as well as in patients with intensive care (ICU) coma without the acute brain complication. Also, more studies are needed in this field [46]. Additional studies are needed to evaluate the role of IONM in ABI, posterior fossa surgery, and spinal surgery [65].

In spinal surgery, MEP was found to be more sensitive and specific than SSEP in detecting nerve damage. Combined use of MEP and SSEP provided more valuable information compared to individual use. However, there were differing opinions on the optimal use of these technologies. For example, results of Azad et al.'s study showed that patients with MEP changes were 56 times more likely to have postoperative defects [49]. Also, the efficiency of single and combined use of MEP and SSEP was examined in other studies. For example, Thirumala et al. Fehlings et al. and Di Martino et al. reported the advantages of combined use of MEP and SSEP compared to the use of these technologies individually as a valid diagnostic tool in measuring nerve damage [51, 54, 55]. However, Ajiboye et al. reported that using the technology individually has a better effect compared to combined use [52]. Also, Rijs et al. suggested that large prospective studies are needed to gain more knowledge about the value of combined use of MEP and SSEP [48].

Overall, neuromonitoring technologies were found to be useful in identifying nerve damage in various surgical procedures. However, due to heterogeneity and lack of strong clinical evidence, further research is needed to accurately determine the clinical value of neuromonitoring and define appropriate warning criteria [59].

## 5 Limitations

The limitations of this study include the lack of strong evidence and the nonrandom nature of the included studies, which limit the generalizability of the conclusions. The challenges surrounding the use of neuromonitoring technology also pose limitations [59, 61]. Additionally, the quality of the included studies in the meta‐analyses was not evaluated, and the reliance on existing meta‐analyses rather than original data studies may impact the conclusions. The possibility of overlap between the studies included in the meta‐analyses was not examined, which could affect the results. The qualitative synthesis of the study results also makes it difficult to establish a causative relationship. Furthermore, the lack of sufficient evidence in some surgeries makes it challenging to draw conclusions about the effectiveness of IONM.

## 6 Conclusion

In conclusion, further prospective studies, large randomized controlled trials, and economic evaluation studies are needed to definitively assess the effectiveness and safety of IONM. The heterogeneity in study types and thresholds for warning criteria, as well as the uncertain benefits of preventing new neurological defects, necessitate further research in this area.

## Author Contributions

**Aziz Rezapour:** conceptualization, methodology, writing – original draft, supervision, project administration. **Naser Derakhshani:** conceptualization, writing – review and editing, methodology, data curation; resources. **Seyedeh Narges Pouyan:** writing – original draft, writing – review and editing; visualization. **Vahid Alipour:** writing – original draft, methodology, resources, data curation. **Seyed Jafar Ehsanzadeh:** writing – original draft, writing – review and editing, data curation. **Arash Zare‐Sadeghi:** conceptualization, methodology, data curation, resources. **Jalal Arabloo:** conceptualization, writing – original draft, writing – review and editing, methodology, project administration, data curation.

## Ethics Statement

The study was approved by the Ethics Committee of the Iran University of Medical Sciences (IR.IUMS.REC.1399.1233). Consent forms was not applicable for this study. The article does not require any human/animal subjects to acquire such approval.

## Consent

The authors have nothing to report.

## Conflicts of Interest

The authors declare no conflicts of interest.

## Transparency Statement

The lead author Jalal Arabloo affirms that this manuscript is an honest, accurate, and transparent account of the study being reported; that no important aspects of the study have been omitted; and that any discrepancies from the study as planned (and, if relevant, registered) have been explained.

## Supporting information

appendix file 1.

appendix file 2.

appendix file 3.

appendix file 4.

appendix file 5.

appendix file 6.

appendix file 7.

## Data Availability

The data that support the findings of this study are available from the corresponding author upon reasonable request.
