# Supplementary material for: Safety and Efficacy of Intraoperative Neuromonitoring: An Umbrella Review
Source: Health Sci Rep. 2025 Oct 13;8(10):e71370. doi: 10.1002/hsr2.71370 (PMC12516239; doi:10.1002/hsr2.71370)
Supplement: Supplementary file 1 — appendix file 1. [file HSR2-8-e71370-s005.docx]

| Appendix File 1: Quality assessment of studies­­ | | | | | | | | | | | | |
| --- | --- | --- | --- | --- | --- | --- | --- | --- | --- | --- | --- | --- |
| Author | Year | Is the review question clearly and explicitly stated? | Were the inclusion criteria appropriate for the review question? | Was the search strategy appropriate? | Were the sources and resources used to search for studies adequate? | Were the criteria for appraising studies appropriate? | Was critical appraisal conducted by two or more reviewers independently? | Were there methods to minimize errors in data extraction? | Were the methods used to combine studies appropriate? | Was the likelihood of publication bias assessed? | Were recommendations for policy and/or practice supported by the reported data? | Were the specific directives for new research appropriate? |
| Zhu et al. | 2019 | Y | Y | Y | Y | Y | Y | Y | Y | Y | Y | Y |
| Zheng et al. | 2013 | Y | N | Y | Y | Y | Y | Y | Y | Y | N | N |
| Yang et al. | 2017 | N | N | U | N | Y | U | U | Y | Y | N | N |
| Wong et al. | 2017 | N | Y | Y | Y | Y | U | U | Y | Y | N | N |
| Thomas& Guo | 2017 | Y | Y | U | Y | Y | U | N | Y | Y | N | N |
| Thirumala et al. | 2017 | Y | Y | Y | N | Y | Y | Y | Y | Y | Y | Y |
| Thirumala et al. | 2016 | Y | Y | Y | N | Y | Y | Y | Y | N | N | N |
| Thirumala et al. | 2016 | Y | Y | Y | N | Y | U | Y | Y | Y | N | N |
| Thirumala et al. | 2016 | Y | Y | Y | N | N | N | Y | Y | N | N | N |
| Thirumala et al. | 2016 | Y | Y | Y | Y | Y | U | Y | Y | Y | N | N |
| Thiagarajan et al. | 2015 | Y | Y | Y | Y | Y | U | Y | Y | Y | N | N |
| Sun et al. | 2017 | Y | Y | Y | Y | Y | U | Y | Y | Y | Y | Y |
| Rijs et al. | 2019 | Y | Y | Y | Y | Y | U | U | Y | Y | Y | Y |
| Reddy et al. | 2018 | Y | Y | Y | Y | N | N | U | Y | N | N | N |
| Higgins et al. | 2011 | Y | Y | Y | Y | Y | U | U | Y | Y | Y | Y |
| Sanabria et al. | 2013 | Y | Y | Y | Y | Y | Y | U | Y | Y | Y | Y |
| Thirumala et al. | 2017 | Y | Y | Y | Y | Y | U | Y | Y | Y | N | N |
| Ajiboye et al. | 2017 | y | y | y | y | y | y | N | y | N | N | y |
| Azad et al. | 2018 | y | y | y | N | y | N | N | y | N | N | y |
| Chan et al. | 2020 | y | y | y | y | y | y | y | y | N | y | y |
| Cirocchi et al. | 2019 | y | y | y | y | y | y | y | y | y | y | y |
| Daniel et al. | 2018 | y | y | y | y | y | N | y | y | N | y | y |
| Di Carlo et al. | 2020 | y | y | y | y | y | y | y | y | y | N | N |
| Acioly et al. | 2013 | y | y | y | N | N | N | N | y | N | N | y |
| Barbosa et al. | 2015 | y | y | y | y | N | N | y | y | N | N | y |
| Claassen et al. | 2014 | y | y | y | N | y | y | y | y | N | y | N |
| D'Amico et al. | 2020 | y | N | y | N | N | N | N | y | N | N | y |
| Fok et al. | 2015 | y | y | y | y | N | N | y | y | N | N | y |
| Fehlings et al. | 2010 | y | y | y | y | y | y | y | y | N | y | y |
| Dionigi et al. | 2017 | N | y | y | y | N | N | y | y | N | N | y |
| Di Martino et al. | 2019 | y | y | y | y | y | N | y | y | N | N | y |
| Devlin et al. | 2006 | y | N | y | N | N | N | n | y | N | N | y |
| Bai and Chen | 2018 | y | y | y | y | y | U | y | y | y | y | y |
| Rulli et al. | 2014 | y | y | y | y | N | N | y | y | y | y | y |
| Holdefer et al | 2020 | Y | Y | N | N | Y | N | N | U | N | y | y |
| Naytah et al | 2019 | Y | Y | Y | Y | Y | U | Y | Y | N | Y | N |
| Ishida et al |  | Y | Y | Y | Y | Y | U | Y | Y | Y | Y | Y |
| Pardal-Refoyo et al | 2016 | Y | Y | Y | Y | N | U | U | Y | U | Y | Y |
| Nasi et al | 2020 | Y | Y | Y | Y | Y | U | Y | Y | Y | Y | N |
| Liu et al | 2017 | Y | Y | N | Y | Y | U | U | N | N | Y | N |
| Maza-Krzeptowsky et al | 2018 | Y | Y | N | Y | Y | N | Y | N | N | Y | Y |
| Mikula et al | 2016 | Y | Y | Y | Y | Y | N | Y | Y | Y | Y | N |
| Malik and Linos | 2016 | Y | Y | Y | Y | Y | U | N | N | N | Y | Y |
| Lombardi et al | 2016 | Y | Y | Y | Y | Y | Y | Y | Y | N | Y | Y |
| Kondziella et al | 2015 | Y | Y | Y | Y | Y | Y | Y | Y | N | Y | Y |
| Pisanu et al | 2014 | Y | Y | Y | Y | N | N | Y | Y | N | Y | Y |
| McGarvey et al. | 2014 | Y | Y | N | Y | Y | N | N | N | N | N | Y |
| Y - Yes, N - No, U – Unclear, NA- Not applicable | | | | | | | | | | | | |

­
