# Supplementary material for: Safety and Efficacy of Intraoperative Neuromonitoring: An Umbrella Review
Source: Health Sci Rep. 2025 Oct 13;8(10):e71370. doi: 10.1002/hsr2.71370 (PMC12516239; doi:10.1002/hsr2.71370)
Supplement: Supplementary file 2 — appendix file 2. [file HSR2-8-e71370-s002.docx]

| **Appendix 2- Features of included studies using IONM during thyroid surgery (thyroidectomy)** | | | | | | | | | | | |
| --- | --- | --- | --- | --- | --- | --- | --- | --- | --- | --- | --- |
| **Author(s)** | **Publication date** | **Study design** | **Objective** | **Searched databases** | **Time interval of included studies** | **Number and type of included studies** | **Quality control** | **Quality assessment tool** | **Analysis** | **Statistical model used** | **Indicators** |
| Cirocchi et al. | 2019 | Systematic review and meta-analysis | Evaluation of the effects of IONM compared with visual identification of the nerve to prevent RILN injury in adults undergoing thyroid surgery. | CENTRAL, MEDLINE, Embase, ICTRP Search Portal and ClinicalTrials.gov. (21 August 2018) | 2013-2018 | Five randomized controlled trial (RCT) studies | Yes | GRADE | Yes | Random effects model | Risk ratio |
| Dionigi et al. | 2017 | Systematic review | Investigation and evaluation of the use of IONM in endoscopic thyroidectomy. | CENTRAL, MEDLINE, Cochrane and EMBASE (from January 1, 2000 to September 1, 2016) | 2000-2015 | Six study (retrospective studies / three prospective randomized studies) | No | No | No | No | None |
| Higgins et al. | 2011 | meta-analysis | Comparison of the effect of recurrent laryngeal nerve (RLN) compared to RLN identification alone on the actual degree of vocal cord paralysis after thyroidectomy. | MEDLINE (1966-July 2008), EMBASE (1980-July 2008), Cochrane Central Register of Clinical Trials (CENTRAL), Cochrane Database of Systematic Reviews, clinicaltrials.gov, and The National Guideline Clearinghouse databases | 1991-2009 | Forty two studies (1 randomized clinical trial, 7 comparative trials and 34 case series) | Yes | CONSORT | Yes | Random effects model | Chance ratio |
| Sanabria et al. | 2013 | meta-analysis | Conducting a meta-analysis of the combined results of individual studies to measure the frequency of RLN and EBSLN injuries in patients undergoing neuromonitoring during thyroidectomy compared to the usual method of identification. | The Cochrane Central Register of Controlled Trials (CENTRAL) on The Cochrane Library (2012), The National Library of Medicine (PubMed) (1966–December 2012), EMBASE (1980–December 2012) and The Latin American and Caribbean Health Sciences Library (LILACS) (1980–December 2012). | 2009-2012 | Six randomized controlled trials | Yes | GRADE | Yes | Random effects model | Risk difference |
| Sun et al. | 2017 | meta-analysis | To evaluate whether IONM can reduce the incidence of RLN palsy to a greater extent than visual identification of RLN alone in thyroid reoperations. | PubMed, SCIE and Wan Fang databases/studies published up to 31 August 2016. | 2004-2014 | Nine studies (2 prospective cohort studies and 7 retrospective cohort studies) | Yes | Newcastle-Ottawa Scale (NOS) | Yes | Random effects model | Risk ratio |
| Wong et al. | 2017 | Systematic review and meta-analysis | To evaluate the role of IONM in reducing RLN paralysis during high-risk thyroidectomy and to identify which high-risk subgroups would benefit the most. | Pubmed, Medline, Embase and Cochrane central register of clinical trials (CENTRAL) from 1st January 2000 to 30th June 2015 | 2004-2014 | Ten studies (2 prospective comparative studies and 8 retrospective studies) | Yes | Newcastle-Ottawa Scale (NOS) | Yes | Fixed effects model | Chance ratio |
| Yang et al. | 2017 | meta-analysis | Using a meta-analysis method to evaluate the role of IONM in assisting thyroid surgery. | PubMed, Embase, and the Cochrane library from January 1, 2004 to July 30, 2016. | 2004-2016 | Twenty four studies (4 randomized controlled trials (RCT), 20 RCS studies). | Yes | Cochrane Collaboration tool | Yes | Random effects model | Chance ratio |
| Zheng et al. | 2013 | meta-analysis | To evaluate the effect of IONM during thyroid surgery. | The web-based PubMed database (1950 through April 2011), Embase (1974 through April 2011), and the Cochrane Central Register of Controlled Trials (CENTRAL, The Cochrane Library, Issue 2 of 4, April 2011) | 1992-2009 | Fourteen studies (2 randomized clinical trials, and 12 comparative trials) | Yes | Cochrane Risk of Bias tool | Yes | Random effects model | Chance ratio |
| Lombardiet al. | 2016 | meta-analysis | Determining the benefit of using intermittent neuromonitoring during surgery to prevent permanent nerve paralysis. | PubMed, Scopus, and Cochrane Central Register of Controlled Trials until August 2014 | 2004-2014 | Fourteen studies (4 randomized controlled trials (RCTs), and 10 non-randomized studies (NRS)) | Yes | Cochrane Collaboration’s tool | Yes | Fixed effects model | Risk ratio/risk difference |
| Naytah et al. | 2019 | Systematic review and meta-analysis | Evaluation of the advantage of IONM in identifying EBSLN during thyroid surgery. | MEDLINE, PubMed, Web of Science, and Cochrane Library January 1, 1995, through July 1, 2018 | 2009-2016 | Seven studies | Yes | Cochrane Collaboration tool | Yes | Random effects model | Risk ratio |
| Pardal-Refoyo et al. | 2016 | Systematic review and meta-analysis | Risk estimation of bilateral RLN paralysis with and without intraoperative neuromonitoring. | PubMed, Scopus (EMBASE) and the Cochrane Library in the period 2000-2014 | 2000-2014 | Forty studies (clinical trials, cohort studies and case series) | No | No | Yes | Random effects model | Incidence rate/adjusted hazard ratio |
| Pisanu et al. | 2014 | Systematic review and meta-analysis | Assessing the potential improvement of IONM versus visual identification of RLN alone (VA) in reducing the incidence of vocal cord paralysis. | Embase, Medline, Cochrane, PubMed, and Google Scholar databases in August 2013 | 2004-2012 | Twenty studies (3 prospective randomized trials, 7 prospective trials and 10 retrospective observational studies) | Yes | MOOSE | Yes | Fixed effects model | Standardized Mean Difference (SMD) |
| Malik and Linos | 2016 | Systematic review | Evaluating the effectiveness of intraoperative neuromonitoring (IONM) in preventing recurrent laryngeal nerve palsy (RLNP) during thyroid surgery. | MEDLINE, EMBASE, and PubMed from 1999 forwards | 2004-2014 | Seventeen studies (12 comparative studies, 1 randomized clinical trial, 2 non-randomized clinical trials and 2 case studies) | No | No | No | Not reported | Not reported |
| Bai and Chen | 2018 | meta-analysis | Determination of the effects of IONM in thyroidectomy. | MEDLINE (PubMed), BIOSIS Previews (ISI Web of Knowledge) and Cochrane library from January 1980 to July 2017 | 2002-2017 | Thirty four studies (3 randomized controlled trials and 31 non-randomized trials) | Yes | Newcastle-Ottawa Scale (NOS) | Yes | Random effects model | Risk ratio/risk difference |
| Rulli et al. | 2014 | meta-analysis | Evaluation of the risk of temporary or permanent RLN damage in thyroid surgery with or without IONM | PubMed and Ovid, and the Cochrane Library database from January1994 to February 2012 | 2002-2009 | Eight studies (2 randomized trials and 6 non-randomized comparative studies). | No | No | Yes | Fixed effects model | Risk ratio |

| **Appendix 2- Summary of the findings of included studies on the use of IONM during thyroid surgery (thyroidectomy)** | | | | | | | | |
| --- | --- | --- | --- | --- | --- | --- | --- | --- |
| **Author(s)** | **Publication date** | **Patient population** | **Sample size** | **Interventions** | **Comparator** | **Outcomes** | **Estimated cumulative total index** | **Main findings** |
| Cirocchi et al. | 2019 | Adults (over 18 years) undergoing thyroidectomy | 1,558 patients / 781 people in the IONM group, and 777 people in the visual nerve identification group alone | IONM | Visual identification of the nerve alone | Primary outcomes: permanent RILN paralysis, temporary RILN paralysis, health-related quality of life  Secondary outcomes: adverse events other than permanent or temporary RILN paralysis, operative time, all-cause mortality, | Stable evidence of advantages or disadvantages of comparing IONM with visual nerve identification alone for permanent RILN palsy (RR: 0.77; 95% CI: 0.33 to 1.77; P = 0.54; 4 trials; 2895 nerves at risk; very low-certainty evidence) or transient RILN palsy (RR: 0.62; 95% CI 0.35 to 1.08; P = 0.09; 4 trials; 2895 nerves at risk; very low-certainty evidence). None of the trials reported health-related quality of life. Transient hypoparathyroidism as an adverse event was not significantly different between the intervention and comparator groups (RR: 1.25; 95% CI 0.45 to 3.47; P = 0.66; 2 trials; 286 participants; very low-certainty evidence). Operative time was comparable between IONM and optic nerve monitoring alone (MD: 5.5 minutes; 95% CI -0.7 to 11.8; P = 0.08; 3 trials; 1251 participants; very low-certainty evidence).  Three of the five included trials provided data on all-cause mortality: no deaths were reported; None of these trials reported socioeconomic effects. The evidence reported in this review was mostly of very low certainty, particularly because of the risk of bias, the high degree of imprecision due to wide confidence intervals, and the significant between-study heterogeneity. | There was no strong evidence on the advantages or disadvantages of using intraoperative neuromonitoring compared with visual nerve identification alone for permanent or temporary recurrent laryngeal nerve palsy, side effects, and operative time. |
| Dionigi et al. | 2017 | Patients undergoing endoscopic or robotic thyroidectomy | Not mentioned | IONM | Direct RLN stimulation / standard endoscopic procedures | Paralysis (RLN) (extent, temporary vs. permanent and unilateral vs. bilateral) | Reports of endoscopic and robotic IONM include their use for revision surgery and use in benign and malignant cases. None of the IONM endoscopic procedures involved bilateral paralysis. The rate of recurrent laryngeal paralysis was 0-3.6% for temporary and 0-0.4% for permanent. | IONM facilitates identification of RLN and SLN, enabling testing of RLN and SLN function. This operation can be corrected in three stages of surgery. |
| Higgins et al. | 2011 | Patients undergoing thyroidectomy | 64,699 nerves at risk | IONM | Visual identification of the nerve alone | Incidence of true vocal cord paralysis (TVFP) | The overall incidence of true vocal cord palsy (TVFP) for neuromonitoring (IONM) was 3.52% versus 3.12% for visual nerve identification (ID) alone (OR 0.93; 95% confidence interval [CI], 0.76–1.12]). There was no statistically significant difference in temporary TVFP (2.74% IONM vs. 2.49% ID [OR 1.07, 95% CI, 0.95–1.20]), permanent TVFP (0.75% IONM vs. 0.58% ID [OR 0.99, 95% CI, 0.79–1.23 ]), or unintentional RLN injury (0.12% IONM vs 0.33% ID [OR 0.50, 95% CI, 0.15–1.75]) was not found. | This meta-analysis shows no statistically significant difference in the rate of vocal cord paralysis after using intraoperative neuromonitoring versus identifying the recurrent laryngeal nerve alone during thyroidectomy. |
| Sanabria et al. | 2013 | Patients undergoing partial or total elective thyroidectomy for benign or malignant disease | 1,602 patients and 3,064 nerves at risk | IONM | Visual identification of the nerve alone | Temporary and permanent paralysis of RLN and EBSLN | The risk difference for temporary RLN paralysis, definitive RLN paralysis, temporary EBSLN paralysis, and definitive EBSLN paralysis was -2% (95% CI -5.1 to 1). 0% (-1 to 1); -9 (-15 to -2) and -1 (-4 to 2). | The meta-analysis did not show a statistically significant reduction in the risk of temporary or permanent RLN injury and definite EBSLN injury with the use of neuromonitoring. The norm-monitoring group had a statistically significant reduction in the risk of temporary EBSLN injury. |
| Sun et al. | 2017 | Patients undergoing re-thyroid surgeries | 2,436 nerves at risk | IONM | Visual identification of the nerve alone | Permanent paralysis of RLN, temporary paralysis of RLN, total paralysis of RLN | The rate of total RLN paralysis in reoperations performed with IONM was significantly lower than those performed without IONM (RR = 0.434). The rate of temporary RLN paralysis with and without IONM was not significantly different (RR = 0.607, 95% CI = 0.270-1.366, P = .227). However, IONM was significantly associated with a reduction in permanent RLN paralysis (RR = 0.426, 95% CI = 0.196–0.925, P = .031). No significant heterogeneity was found (I(2) = 13.7%, P = .325). | IONM is associated with reduced total and permanent RLN paralysis in thyroid reoperation. |
| Wong et al. | 2017 | Patients with the history of thyroid surgery, thyroidectomy for thyroid cancer, thyrotoxicosis, or retrosternal goiter | 4,460 nerves at risk in the VA group, and 6,155 nerves at risk in the IONM group | IONM | Visual identification of the nerve alone | Temporary RLN paralysis, permanent RLN paralysis | Compared with visual identification, IONM had a lower rate of total RLN paralysis [4.5% vs. 2.5%, odds ratio (OR): 1.40, 95% confidence interval (CI): 1.12-1.79, p = 0.003] and temporary [3.9% In contrast, it was 2.4%; OR: 1.47, 95% CI: 1.07–2.00, p = 0.016] in high-risk total thyroidectomies. The use of IONM reduced the rate of total RLN paralysis during reoperation and temporary RLN paralysis during thyroidectomy for malignancy. The use of IONM reduced total RLN palsy during thyroidectomy for malignancy compared to VA (3.5% vs. 2.1%, p = 0.050). | Wong et al. showed that in high-risk thyroidectomies, the use of IONM, especially in the subgroups of reoperation and malignancy, is beneficial in reducing overall RLN. |
| Yang et al. | 2017 | Patients undergoing thyroid surgery | 9,203 patients and 17,203 nerves at risk | IONM | Visual identification of the nerve alone | Permanent paralysis of RLN, temporary paralysis of RLN, total paralysis of RLN | The incidence of general, temporary, and permanent RLNP in the IONM group was 15.3%, 82.1%, and 67.0%, respectively, while for the visual nerve identification group, it was 37.4%, 58.2%, and 17.0%. It was % percent. The summary ratio of overall, temporary and permanent RLNP index using IONM and visual identification was 0.81 (95% CI 0.66-0.99), 0.76 (95% CI 0.61-0.94) and 0.78 (95% CI 0.55-1.09), respectively. | The presented data showed benefits in reducing the rate of RLNP using IONM, but no statistically significant difference for the rate of permanent RLNP. |
| Zheng et al. | 2013 | Patients undergoing thyroidectomy | 36,487 nerves at risk | IONM | Visual identification of the nerve alone | Temporary paralysis of RLN, total paralysis of RLN | There was a statistically significant difference in total RLN palsy (3.37% with intraoperative neuromonitoring [IONM] vs. 3.76% without IONM [OR: 0.74; 95% confidence interval [CI]: 0.59-0.92]) and temporary RLN palsy ( 2.56% were identified with IONM versus 2.71% without IONM [OR: 0.80; 95% CI: 0.65–0.99]). The incidence of permanent RLN palsy was 0.78% for IONM versus 0.96% for visual identification of the nerve (OR: 0.80; 95% CI: 0.62–1.03). | Based on this meta-analysis, there were statistically significant differences in the incidence of total and temporary recurrent laryngeal nerve palsy after the use of IONM versus identification of the recurrent laryngeal nerve alone during thyroidectomy. However, there was no statistically significant difference in the incidence of persistent interlaryngeal nerve palsy between the groups. |
| Lombardiet al. | 2016 | Patients undergoing thyroid surgery | 32,290 nerves at risk | IONM | Visual identification of the nerve alone | Permanent injuries of RLN | According to meta-analyses, including nonrandomized studies that persisted as a permanent injury for 6 months or 12 months after thyroid surgery, the overall relative risks were 0.79 (95% confidence interval, 0.60–1.05) and 0.75 ( CI, 0.60–1.05), respectively. 95%, 0.43-1.30). As for the meta-analysis including randomized controlled trials, the risk difference was 0.00 (95% confidence interval, 0.01 to 0.00). No heterogeneity was observed in any of the analyzes performed (Higgins index = 0). | Results show that the use of IONM does not prevent permanent nerve palsy. No significant advantage of IONM over visual identification alone in reducing the rate of recurrent laryngeal nerve injury has been demonstrated. Consequently, IONM should not be considered standard of care in preventing RLN paralysis. |
| Naytah et al. | 2019 | Adult patients (over 18 years old) who have had thyroid surgery, and attempts being conducted to identify EBSLN with conventional methods and/or IONM | 726 patients in the neuromonitoring group,  and 730 control patients | IONM | Conventional method (dissection) | Number of EBSLNs at risk and number of EBSLNs identified/visualized | Statistical analysis showed a significant improvement in EBSLN visualization in the monitoring group  (RR = 0.7245, 95% CI = 0.3649, 1.0840, P < 0.0001). | The use of IONM during open thyroid surgery increases the identification/visualization of the EBSLN and hence may reduce the incidence of voice disturbances after thyroidectomy. |
| Pardal-Refoyo et al. | 2016 | Patients with total thyroidectomy | 30,922 patients | IONM | Without IONM | Prevalence of bilateral recurrent laryngeal nerve damage | The prevalence of BLP was lower in the series with neuromonitoring compared to without neuromonitoring (2.43 ‰ [1.55 --- 3.5 ‰] vs. 5.18 ‰ [2.53 --- 8.7 ‰]). This difference is equivalent to an absolute risk reduction of 2.75‰ with a number needed to treat of 364.13. The neuromonitoring group was more homogeneous (I 2 = 7.52%) than those without NM (I 2 = 79.32%). The difference observed in the subgroup analysis was incorrect because the number of paralysis observed was too small. | The risk of bilateral paralysis is lower in studies with neuromonitoring. |
| Pisanu et al. | 2014 | Patients undergoing thyroidectomy | 23,512 patients,  35,513 nerves at risk | IONM | Visual identification of the nerve alone | Permanent paralysis of RLN, temporary paralysis of RLN, total paralysis of RLN | The total number of nerves at risk was 35,513, with 24,038 nerves (67.7%) in the IONM group and 11,475 nerves (32.3%) in the visual nerve identification (VA) group. The rate of total RLN paralysis per nerve at risk was 3.47 in the IONM group and 3.67 in the VA group. The rate of temporary RLN paralysis per nerve at risk was 2.62% in the IONM group and 2.72% in the VA group. The rate of permanent RLN palsy per nerve at risk was 0.79% in the IONM group and % in the VA group. None of these differences were statistically significant and no other differences were found. | The present review with meta-analysis showed that there is no statistically significant difference in the incidence of RLN paralysis when using IONM compared to visual identification of the nerve during thyroidectomy. |
| Malik and Linos | 2016 | Patients undergoing thyroid surgery with  or without IONM | 30,926 patients | IONM | Without IONM | Total RLN paralysis per nerve at risk | Total RLN paralysis per nerve at risk for IONM group, control group was 3.18% and 3.83%, respectively. | There is no statistically significant difference between IONM and control. |
| Bai and Chen | 2018 | Patients undergoing thyroidectomy | 59,380 nerves at risk | IONM | Without IONM | Permanent paralysis of RLN, temporary paralysis of RLN, total paralysis of RLN | Overall analysis showed a significant reduction in overall damage (RR = 0.68, 95 C CI: 0.55 to 0.83), temporary damage (RR = 0.71, 95 C CI: 0.57 to 0.88), and permanent RLN damage (RD = −0.0026, 95) with IONM (CI: −0.0039 to −0.0012). Subgroup analysis showed that IONM in patients undergoing bilateral thyroidectomy has a preventive role of overall, temporary and permanent damage. IONM also reduced overall and temporary damage rates for malignant cases. Surgery with IONM was associated with less total and temporary RLN injuries at a surgical volume of less than 300 nerves at risk per year and less total and permanent RLN injuries at a surgical volume of more than 300 nerves at risk per year. | The use of IONM can reduce thyroidectomy RLN injury. In particular, we recommend routine IONM for use in bilateral surgeries and surgeries for malignancies and cancers. |
| Rulli et al. | 2014 | Total, subtotal or near-total thyroidectomy and thyroid lobectomy in patients with thyroid cancer or benign thyroid disease | 3,029 patients and 5,257 nerves at risk | IONM | Without IONM | Permanent paralysis of RIN, temporary paralysis of RIN | IONM showed a significant effect in preventing temporal injuries (positive predictive value = 5% [95% CI: 2-8], negative = 96% [95% CI: 91-100], relative risk = 0.73 [95% CI: 0.54)–0.98], p = 0.035), while no effect was shown on permanent damage (positive predictive value = 2% [95% CI: 0.6–3.8], negative 99% [95% CI: 97–100 ], and relative risk = 0.73 [95% CI: 0.44–1.23], p = 0.235).) | This meta-analysis showed the effect of IONM in preventing temporary injury during thyroidectomy. No benefit was seen in permanent damage. |
