# Supplementary material for: Safety and Efficacy of Intraoperative Neuromonitoring: An Umbrella Review
Source: Health Sci Rep. 2025 Oct 13;8(10):e71370. doi: 10.1002/hsr2.71370 (PMC12516239; doi:10.1002/hsr2.71370)
Supplement: Supplementary file 3 — appendix file 3. [file HSR2-8-e71370-s007.docx]

| **Appendix 3- Characteristics of included studies using IONM during brain aneurysm surgery** | | | | | | | | | | | |
| --- | --- | --- | --- | --- | --- | --- | --- | --- | --- | --- | --- |
| **Author(s)** | **Publication date** | **Study design** | **Goal** | **Searched data bases** | **Time interval of included studies** | **Number and type of included studies** | **Quality control** | **Quality assessment tool** | **Analysis** | **Statistical model used** | **Indicator** |
| Nasi et al. | 2020 | Systematic review and meta-analysis | To evaluate whether IOM can prevent nerve damage during clipping of intracranial aneurysm. | PubMed/Medline, Scopus, and Cochrane databases. January 1999 and January 2019 | 2010-2019 | Four studies (3 included retrospective cohort studies (before and after IOM use) and 1 prospective cohort study to evaluate IOM changes during MCA aneurysm surgery) | Yes | MINORS | Yes | Random effects model | Chance ratio |
| Kondziella et al | 2015 | Systematic review | Evaluation: (a) diagnostic accuracy of cEEG as a confirmatory test, (b) prognostic value of EEG patterns indicative of seizures and DCI, and (c) efficacy of neuromonitoring using cEEG in terms of improving clinical outcome following SAH. | Cochrane Central Register of Controlled Trials (The Cochrane Library), Medline (PubMed), EMBASE, Scopus,  from January 1, 1980 to June 15,2014 | 1991-2014 | Eighteen studies (single center case series (including a randomized clinical trial) | Yes | QUADAS-2 | No | _ |  |
| Thomas& Guo | 2017 | Systematic review and meta-analysis | Investigating the diagnostic accuracy of different evoked potential monitoring techniques in predicting postoperative neurological deficits in brain aneurysm surgery. | MEDLINE, Embase and Cochrane databases 1983 through March 2016 | 1994-2013 | Fifteen prospective studies | Yes | QUADAS | Yes | Random effects model |  |
| Zhu et al. | 2019 | Meta-analysis | To assess the accuracy of the intraoperative evoked potential (EP) diagnostic test (DTA) for the diagnosis of brain injury during brain aneurysm surgery. | EDLINE, EMBASE, LILACS, IndMed and a variety of other sources from 1 January 1960 to 5 January 2016 (last updated on 27 June 2018) | 1987- 2017 | Thirty five observational studies (prospective and retrospective clinical studies) | Yes | QUADAS-2 | Yes | Random effects model |  |
| Fok et al. | 2015 | Systematic review | Evaluation of the benefits and application of neuromonitoring in descending aneurysm treatment surgery and thoracoedema (DTA and TAAAR). | OVID Medline, PUBMED, Scopus, and COCHRANE (from their dates of inception until February 2014) | 1999- 2013 | Fifteen prospective and retrospective studies | No | _ | No | _ | _ |

| **Appendix 3- Summary of the findings of the included studies on the use of IONM during brain aneurysm surgery** | | | | | | | | |
| --- | --- | --- | --- | --- | --- | --- | --- | --- |
| **Author(s)** | **Publication date** | **Patient population** | **Sample size** | **Interventions** | **comparator** | **Outcomes** | **Estimated cumulative total index** | **Main findings** |
| Nasi et al. | 2020 | Patients undergoing brain aneurysm surgery | 873 patients | IONM | Without IONM | Nerve damage during brain aneurysm surgery | Patients who underwent surgery with IONM had less neurological deficit than those who underwent surgery without it (odds ratio [LOR] -0.84 -1.64; -0.03]; p = 0.01 04). There were also fewer neurologic events in patients under monitoring without statistical significance (LOR for permanent deficit was -0.82 [-1.65; -0.00]; p = 0.05. | The use of IONM during brain aneurysm surgery was associated with a reduction in new neurological deficits. However, at long-term follow-up, the use of IOM was not associated with significant improvement in neurologic outcomes. |
| Kondziella et al | 2015 | Patients with aneurysmal subarachnoid hemorrhage (SAH) undergoing cerebral aneurysm surgery | 481 patients with aneurysm SAH | cEEG | Without IONM |  | _ | cEEG monitoring following SAH detects a greater number of subclinical seizures and may predict DCI hours in advance. NCSE is associated with high mortality and morbidity, while for DCI detected by cEEG this association is less clear. |
| Thomas& Guo | 2017 | Patients undergoing brain aneurysm surgery | 1597 patients, 1689 aneurysms | SSEP, TcMEP, DcMEP | Different techniques for monitoring evoked potentials | Sensitivity and specificity | Intervariate sensitivity and specificity for SSEP were 48% (95% confidence interval [CI], 30.7–65.0%) and 92% (CI, 88%–94.4%), 73% (CI, 21.0%–96.7%), and 94%, respectively. (CI, 87.1%–97.5%) for TcMEP; and it was 97% (CI, 74.43%–99.99%) and 89% (CI, 84.0%–94.5%) for DMEP. ROC curve analysis showed that TcMEP had the highest accuracy (area under the ROC curve 0.95; 95% CI, 0.93–0.97), followed by DMEP (0.91, 0.89–0.94) and SSEP (0.88, 0.85–0.91). | TcMEP and DMEP have higher diagnostic accuracy than SSEP in predicting postoperative neurological deficit. |
| Zhu et al. | 2019 | Adult patients (over 18 years old) with brain aneurysm under general anesthesia, undergoing brain aneurysm surgery | 4,011 | Using the EP method (SSEP, MEP, BAEP) or any combination of these methods | Initial postoperative physical examination with confirmatory radiologic imaging (eg, CT or MRI) | Sensitivity and specificity | Cumulative sensitivity and specificity for predicting neurological deficits were 59% and 86% for SSEP, 81% and 90% for MEP, and 92% and 88% for combined monitoring of SSEP and MEP, respectively. | Due to the moderate quality and high heterogeneity of the available primary studies, the diagnostic value of EP monitoring in brain aneurysm surgery cannot be supported or rejected with certainty. However, the combination of SSEP and MEP seems to provide the best DTA for predicting postoperative stroke. |
| Fok et al. | 2015 | Patients under DTA and TAAAR | 1,297 patients | MEP | _ | Death. Immediate neurological deficit | _ | Hospital mortality was low (6.9). Immediate neurological deficit was low (3.5%). |
