# Supplementary material for: Safety and Efficacy of Intraoperative Neuromonitoring: An Umbrella Review
Source: Health Sci Rep. 2025 Oct 13;8(10):e71370. doi: 10.1002/hsr2.71370 (PMC12516239; doi:10.1002/hsr2.71370)
Supplement: Supplementary file 4 — appendix file 4. [file HSR2-8-e71370-s004.docx]

| **Appendix 4- Characteristics of included studies using IONM during carotid endarterectomy, glioma surgery, cardiac/non-cardiac/thoracic surgery, skull base tumor surgery, posterior fossa surgery, acute brain injury, and brain status during anesthesia** | | | | | | | | | | | |
| --- | --- | --- | --- | --- | --- | --- | --- | --- | --- | --- | --- |
| **Type of surgery** | **Author(s)** | **Publication date** | **Design of the study** | **Objective** | **Searched databases** | **Time interval of included studies** | **Number and type of included studies** | **Quality control** | **Quality assessment tool** | **Analysis** | **Statistical model used** |
| Carotid endarterectomy | Reddy et al. | 2018 | Meta-analysis | Evaluating the efficacy of intraoperative SSEP change in predicting the risk of stroke in the postoperative period, more than 24 hours but within 30 days. | PubMed, Web of Science, and Embase | 1985 -2015 | Twenty-five prospective and retrospective studies | NO | NO | Yes | Bivariate normal model |
| Carotid endarterectomy | Thiagarajan et al. | 2015 | Meta-analysis | Determining whether multimodal monitoring leads to increased diagnostic sensitivity and accuracy. | Embase, PubMed and Web of science databases from 1945 through 26th March 2015 | 1995-2012 | Four prospective and retrospective studies | Yes | QUADAS 2 | Yes | Bivariate model |
| Carotid endarterectomy | Thirumala et al. | 2016 | Meta-analysis | Determine the diagnostic accuracy of electroencephalogram (EEG) in predicting postoperative strokes through a meta-analysis of existing literature. | PubMed and Web of Science databases for relevant literature from 1945 through 8 August 2014 | 1975- 2007 | Thirty prospective and retrospective clinical trial studies | Yes | QUADAS | Yes | Bivariate model |
| Glioma surgery | Di Carlo et al. | 2020 | Systematic review and meta-analysis | Investigation of early and permanent postoperative defects in patients who underwent insular glioma surgery using awake craniotomy with direct electrical stimulation (DES) versus surgery under general anesthesia. | PubMed, Ovid MEDLINE, and Ovid EMBASE (January 1990 to January 2018) | 1997-2016 | Five prospective studies / 3 retrospective studies | Yes | Newcastle-Ottawa Scale | Yes | Random effects model |
| Glioma surgery | Barbosa et al. | 2015 | Systematic review | Investigating the effect of assistive technologies during surgery on the rate of risk (EOR) in glioma surgery, compared to conventional unassisted surgery. | MEDLINE (PubMed), Scopus, Web of Science, and SciELO (2006 -2014) | 2006 - 2013 | Six prospective controlled studies | No | No | No | No |
| Cardiac/non-cardiac/chest surgery | Luo, C. | 2018 | Systematic review and meta-analysis | Evaluating the effectiveness of brain monitoring of the depth of anesthesia in reducing postoperative cognitive function and postoperative delirium. | MEDLINE, EMBASE, and Cochrane Library databases NA | 2011-2013 | Five clinical trial studies | No | No | Yes | Random effects model |
| Tumor surgery of skull base and Cerebellopontine Angle | Acioly et al. | 2013 | Systematic review | Reviewing the current literature emphasizing all aspects of FN monitoring for Cerebellopontine Angle and skull base tumors from description to recent success in predicting the performance of standard and emerging monitoring methods. | PubMed (up to February, 2011) | DES: (1979 -2010) free-running EMG: (1987-2010) FMEP: (2001-2011) | In DES, 27 retrospective studies and 35 prospective studies / in EMG, six retrospective studies and seven prospective studies / in FMEP, three retrospective studies and three prospective studies | No | No | No | No |
| Posterior fossa surgery | D'Amico et al. | 2020 | Systematic review | The review of the available evidence has been carried out with the aim of investigating the neural circuits involved in the pathophysiology of Cerebellar mutism syndrome. | PubMed central (Searching Date Not Mentioned) | Not reported | Two studies (type of study were not mentioned) | Not reported | Not reported | Not reported | Not reported |
| Acute brain injury | Claassen et al. | 2014 | Systematic review | Determining the optimal use and indications of electroencephalography (EEG) in the intensive care management of acute brain injury (ABI). | PubMed (January 1990 through August 15, 2013) | 1979-2013 | Three clinical trial studies, one case study, 80 prospective observational studies, 78 retrospective studies | Yes | GRADE | No | No |
| The state of the brain during anesthesia | Chan et al. | 2020 | Systematic review and meta-analysis | Whether anesthesia management by processed EEG will reduce the rate of unwanted consciousness with recall during anesthesia, postoperative delirium, postoperative neurocognitive disorders and finally long-term mortality after surgery, or not. | Ovid MEDLINE/ EMBASE/Cochrane Central Register of Controlled Trials/ Cochrane Database of Systematic Reviews / PubMed/ Web of Science (2000 to October 1, 2018) | 2003-2019 | Fifteen clinical trial studies | Yes | GRADE | Yes | Random effects model |
