# Supplementary material for: Safety and Efficacy of Intraoperative Neuromonitoring: An Umbrella Review
Source: Health Sci Rep. 2025 Oct 13;8(10):e71370. doi: 10.1002/hsr2.71370 (PMC12516239; doi:10.1002/hsr2.71370)
Supplement: Supplementary file 5 — appendix file 5. [file HSR2-8-e71370-s006.docx]

| **Appendix 5- Summary of the findings of included studies on the use of IONM during carotid endarterectomy, glioma surgery, cardiac/non-cardiac/thoracic surgery, skull base tumor surgery, posterior fossa surgery, acute brain injury, and brain status during anesthesia** | | | | | | | | |
| --- | --- | --- | --- | --- | --- | --- | --- | --- |
| **Type of surgery** | **Author(s)** | **Patient population** | **Sample size** | **Interventions** | **Comparator** | **Outcomes** | **Estimated cumulative total index** | **Main findings** |
| Carotid endarterectomy | Reddy et al. 2018 | Asymptomatic or symptomatic patients who undergo carotid endarterectomy surgery | 8,307 | Somatosensory evoked potentials (SSEPs) | Not reported | Prediction of stroke risk | The diagnostic chance ratio for the period surrounding surgery, i.e. more than 24 hours and within 30 days, was around 8.68. Therefore, the diagnostic chance ratio for unstable and transient SSEP changes and stroke was around 3.88 and for stable SSEP changes and stroke was around 49.29. These ratios for the lack of unstable and transient SSEP changes and stroke and the lack of stable SSEP changes and stroke were reported as 36.45 and 281.35, respectively. | Patients with SSEP changes over the entire 30-day period are at risk of postoperative stroke. There was a significant increase in stroke risk with the severity of SSEP changes. This issue is important from the point of view that SSEP changes can be considered as a predictor of stroke in the 30-day postoperative period during carotid endarterectomy. |
| Carotid endarterectomy | Thiagarajan et al. 2015 | Patients with carotid stenosis (CS) undergoing CEA | 1,970 | Multimodal neurophysiological monitoring | Single neurophysiological monitoring electroencephalography (EEG) and SSEP | Sensitivity, specificity, and diagnostic risk ratio | The diagnostic chance ratio of dual modality neuromonitoring was equal to 17.4. The specificity of simultaneous EEG and SSEP changes in predicting postoperative strokes was calculated at 96.8% (95% confidence interval between 94.1% and 98.3%). The sensitivity of combined monitoring with changes in both methods was significant and equal to 58.9 (95% confidence interval, between 41.2% and 74.7%). | Multimodality monitoring with changes in either EEG or SSEP as warning criteria was 1.32 times more sensitive than EEG alone and 1.26 times more sensitive than SSEP alone. |
| Carotid endarterectomy | Thirumala et al. 2016 | Adults (18 years) who underwent CEA for documented (symptomatic/asymptomatic) carotid stenosis | 8,765 | Electroencephalogram | Not reported | Sensitivity, specificity, and diagnostic risk ratio | Postoperative stroke rate for the cohort of 8765 patients was about 1.75%. The cumulative sensitivity and specificity of EEG changes in predicting these strokes were reported as 52% (with a 95% confidence interval between 43 and 61%) and 84% (with a 95% confidence interval between 81 and 86%), respectively. The summary of the estimates obtained from the subgroups also showed similar results. Also, the diagnostic odds ratio was equal to 5.85 (in the 95% confidence interval, between 3.71 and 9.22%). In terms of the observed stroke rate, the positive odds ratio was 3.25, while the negative predictive value was 98.99%. | Monitoring through EEG has a high specificity in predicting strokes around carotid endarterectomy surgery. |
| Glioma surgery | Di Carlo et al. 2020 | Patients who underwent insular glioma resection. In general, the average age of the patients was 42.3 ± 23.2 years and the proportion of the mentioned patients was 58.8%. | 227 | Direct Electrical Stimulation (DES) or Awake DES | Surgery under general anesthesia | The rate of early and permanent complications after surgery | In general, the rates of initial and permanent failures after the operation were 33.6% and 10.6%, respectively. The rate of early postoperative failures after awake surgery with DES has been higher compared to general anesthesia. However, the rate of permanent postoperative failure in the awake DES subgroup had a lower rate. No significant relationship was observed between the rate of postoperative failures and the amount of neurophysiological monitoring during surgery, intraoperative navigation, year of study and tumor histology. | Awake craniotomy with direct electrical stimulation during insular glioma surgery is associated with a significant reduction in permanent neurological deficits compared with surgery under general anesthesia. Although the high rate of early neurological deficits can be worrisome, most are temporary and resolve weeks after surgery. Accordingly, although more prospective studies are needed, these data support the use of awake VH mapping in insular glioma resection. |
| Glioma surgery | Barbosa et al. 2015 | 726 participants with KPS ≥70 that were suspected newly diagnosed untreated glioma | 726 | Assistive technologies during surgery | Conventional non-assisted surgery | Functional status and progression-free survival | According to the findings, there is strong evidence that the use of 5-ALA is effective for improving EOR. The results of the study on the effect of intraoperative neuromonitoring in high-grade glioma surgery show that overall resection was achieved in 14 of 20 case samples (70%) and 15 of 20 control samples (75%). There was no significant difference between the groups (p=0.67). Observational studies conducted in the field of IONM evaluation in glioma surgery have shown improved EOR for both low-grade and high-grade glioma. The only prospective controlled study showed that in IONM-assisted surgery, the EOR rate for tumors in eloquent regions was not significantly different from the EOR for tumors in non-eloquent regions. |  |
| Cardiac/non-cardiac/chest surgery | Luo, C.2018 | The patients of the two studies underwent non-cardiac surgery, such as hip fracture repair, and the other patients underwent cardiac and thoracic surgery. | 2,868 | Anesthesia based on electroencephalogram | Anesthesia with routine care | Cognitive function | Using a fixed effects model for patients undergoing non-cardiac surgery and a random effects model for cardiac and thoracic surgeries, authors estimated that the cumulative odds ratio for non-cardiac surgery and cardiac surgery significantly decreased the risk of progressive POD in There has been communication. In addition, after dividing the treatment groups into BIS and AEP groups, no significant difference in POD risk reduction was observed between BIS and AEP groups. Significant heterogeneity was observed in AEP studies to indicate the depth of anesthesia. | In summary, significant heterogeneity was identified in the data for cardiac and thoracic surgery, but the overall OR was not significantly different for the two types of surgery. |
| Tumor surgery of skull base and Cerebellopontine Angle | Acioly et al. 2013 | Patients undergoing CPA surgery | Predictive value of DES/HVCA/Predictive value of continuous EMG/Predictive value of FMEP | DES, free-running EMG, FMEP | Not reported | Performance prediction | In large-sized tumors, monitoring during surgery has a good effect on preserving the function and anatomy of the facial nerve. Therefore, neuromonitoring during surgery is considered as one of the modern neurosurgery methods that can improve surgical results by reducing morbidity. The important point in this field is the lack of randomized controlled clinical trial studies on the effect of neuromonitoring during surgery in facial nerve surgery (IOFNM). In addition, IOFNM may lead to improved hearing preservation outcomes by reducing surgical trauma. | Although there is general agreement on the satisfactory performance prediction of various electrophysiological measures, the lack of standardization in electrode assembly and stimulation parameters prevents definitive conclusions about the best method. In addition, studies that emphasize the comparison between criteria or even multimodal monitoring and its effect on anatomical and functional preservation of FN are still missing in the literature. |
| Posterior fossa surgery | D'Amico et al. 2020 | Children with cerebellar mutism (CM) | Not reported | IONM | Not reported | Neurophysiological aspects of the pathophysiology of cerebellar mutism |  | Only two studies have investigated the use of neurophysiology during cerebellar surgery, which indicated a possible somatotopic motor organization of the cerebellar cortex. In addition, extra-operative studies using transcranial magnetic stimulation show the possibility of modulating and measuring the output of the primary motor cortex using a suitable cerebellar stimulator. Theoretically, preservation of this inhibitory or facilitatory modulation may predict preservation of this pathway, while loss of effect may indicate damage to the pathway and predict cerebellar mutism. Similarly, in the extra-operative setting, preoperative and postoperative comparison of transcranial magnetic stimulation of the cerebellum may predict the onset of CM whenever a preexisting modulatory effect is lost as a result of surgery. In fact, there are no data on neurophysiology during cerebellar surgery. This limited knowledge, however, presents a unique opportunity for pediatric neurosurgeons to develop and test existing hypotheses surrounding the pathophysiology of CM, using IONM. |
| Acute brain injury | Claassen et al. 2014 | Patients with severe brain damage | 3,468 | EEG | Continuous EEG monitoring | Diagnosis of ischemia in patients with SAH |  | 1. EEG is recommended for all patients with severe brain injury and persistent, unexplained consciousness (strong recommendation).  2. Urgent EEG is recommended in patients with cSE who do not return to their initial state of function within 60 minutes after taking seizure medication, and urgent EEG (within 60 minutes) is recommended in patients with refractory SE (recommendation Strong).  3. EEG is recommended during hypothermic therapy and within 24 hours of rewarming to rule out NCSz in all comatose patients after cardiac arrest (CA) (strong recommendation).  4. EEG is recommended to rule out NCSz in comatose ICU patients without primary acute brain disease and with unexplained mental status disturbance or neurologic deficit, especially in those with severe sepsis or renal/hepatic failure (recommendation weak).  5. EEG is recommended for diagnosis of delayed cerebral ischemia (DCI) in comatose SAH patients in whom neurological examination is invalid (weak recommendation).  6. Continuous EEG monitoring is recommended as the preferred method over routine EEG monitoring if possible in comatose ICU patients without primary acute brain conditions and with unexplained mental status disturbance or unexplained neurological deficit to exclude NCSz (weak recommendation) ). |
| The state of the brain during anesthesia | Chan et al. 2020 | Patients undergoing surgery under general anesthesia using one of the EEG devices known for monitoring during general anesthesia | 41,509 | EEG monitoring | Routine or standard care | Rates of involuntary consciousness, postoperative delirium, neurocognitive impairment, and long-term mortality after surgery | Findings of the study in the field of unwanted consciousness: The cumulative analysis in this study showed that anesthesia based on BIS will not reduce the risk of consciousness. In patients who received intravenous anesthesia (n = 6283), pooled analysis showed that BIS-based anesthesia significantly reduced the risk of consciousness. BIS monitoring has not created benefit in people who have been under volatile based anesthesia. In trials that compared BIS-based anesthesia with routine care as a control group (n = 17,514), the risk of consciousness was reduced with BIS anesthesia. Excluding trial studies that used patients at high risk of consciousness, the benefits of BIS monitoring have been significant. However, BIS-based anesthesia did not affect consciousness when the active control group (ETAG guided) was used. Findings of the study in the field of postoperative delirium: The overall incidence of postoperative delirium in clinical trials was 22.5% (875 out of 3891). According to the study findings, EEG (or BIS) monitoring reduced the risk of delirium, but there was significant heterogeneity (I2 = 70.8%). The effect of BIS monitoring on relaxation was lost after removing trials. Findings about post-operative neurocognitive disorders: In general, 7.9% of patients had positive neurocognitive disorders for more than 12 weeks after non-cardiac surgery. Cumulative analysis in this study showed that there was a significant increase in the risk of postoperative neurocognitive disorders with BIS monitoring. Results on long-term mortality: In general, deep and light anesthesia based on EEG monitoring had no effect on long-term mortality and significant heterogeneity was observed (I2 = 38.4%). The recommendations of this study were as follows: 1. Doctors are advised to use EEG monitoring to inform anesthesia management. (Weak recommendation, grade D evidence). 2. Clinicians are advised to be knowledgeable in EEG interpretation when using these technologies in anesthesia management (strong recommendation, grade C evidence). 3. To reduce the risk of consciousness with recall in patients undergoing general anesthesia, the use of end-tidal anesthesia monitoring with alarms or processed EEG is recommended (strong recommendation, grade C evidence). 4. To reduce the risk of consciousness with recall in patients receiving intravenous anesthesia during general anesthesia, authors recommended the use of processed EEG monitoring (strong recommendation, grade C evidence). 5. The evidence for the use of processed EEG monitoring in patients with high surgical risk who are under general anesthesia is insufficient in order to reduce the risk of postoperative delirium. 6. Doctors are advised to use EEG monitoring to detect unintentional burst suppression during general anesthesia. (Strong recommendation, grade C evidence). 7. There is insufficient evidence to recommend the use of processed EEG to reduce the risk of postoperative neurocognitive impairment in elderly patients undergoing major non-cardiac surgery. | EEG monitoring should be considered as part of vital organ monitoring to guide anesthesia management. EEG-guided anesthesia reduces the level of consciousness during intravenous anesthesia and has a similar effect in preventing consciousness compared to end-tidal anesthetic gas monitoring. However, there is insufficient evidence to recommend the use of EEG monitoring to prevent postoperative delirium, neurocognitive impairment, or postoperative mortality. Overall, the vigilance rate was low (0.23%, 81 events). |
