# Supplementary material for: Safety and Efficacy of Intraoperative Neuromonitoring: An Umbrella Review
Source: Health Sci Rep. 2025 Oct 13;8(10):e71370. doi: 10.1002/hsr2.71370 (PMC12516239; doi:10.1002/hsr2.71370)
Supplement: Supplementary file 6 — appendix file 6. [file HSR2-8-e71370-s001.docx]

| **Appendix 6- Characteristics of included studies using IONM during spine surgeries** | | | | | | | | | | | |
| --- | --- | --- | --- | --- | --- | --- | --- | --- | --- | --- | --- |
| **Type of surgery** | **Author(s)** | **Date of publication** | **Type of study** | **Goal** | **Searched databases** | **Time interval of included studies** | **Number and type of included studies** | **Quality control** | **Quality assessment tool** | **Analysis** | **The statistical model used in the analysis** |
| Spine deformity surgery | Holdefer et al. | 2020 | Meta-analysis and structural causality model | Improving MEP performance in deformity surgeries considering potential confounders | Embase  from inception to January 2019 | 2002-2019 | Twenty one cohort studies | No | - | Yes | Structural causal model (SCM) |
| Pedicle screw placement | Mikula et al. | 2016 | Systematic review and meta-analysis | Determining the ability and reliability of tEMG technology to identify malpositioned pedicle screws. | the US National Library of Medicine, the Web of Science Core Collection database, and the Cochrane Central Register of Controlled Trials for PS studies | 1994-2014 | Twenty six studies | Yes | the Downs and Black checklist | Yes | In the performed meta-analysis, sensitivity, specificity, overall, and subgroup ROC AUC were calculated.  Publication bias was also assessed through 1-tailed funnel plots. |
| Spinal cord tumor surgery Intra- and extra-modular | Ishida et al. | 2019 | Single-center retrospective cohort and meta-analysis | Summary of clinical outcomes of IONM in patients with ID-EM spinal tumors | PubMed, Embase, Web of Science, and Scopus databases January, 1980, to September 6, 2018 | 2015-2010 | Five retrospective studies | No | Duval and Tweedie’s trim and fill test (PUBLICATION BIAS) | Yes | Pooled diagnostic accuracy was assessed with a 95% confidence interval. Random effects model was also used to estimate the cumulative diagnostic accuracy. Publication bias was assessed by drawing funnel plots, where the x- and y-axes were the logit of event rates and standard errors of each study, respectively, as well as Duval and Tweedie's trim and fill test to determine the number of potentially missing articles and the diagnostic value. Meanwhile, adjusted diagnostic value was used. |
| Cervical decompression surgery | Di Martino et al. | 2019 | Systematic review | Reviewing the evidence related to the use of technology in cervical decompression surgery in the degenerative environment and trying to identify the best supported applications. | PubMed and MEDLINE databases and Cochrane Central Registry of Controlled Trials (from March to July 2017) | 2017-2004 | Eight [Randomized Controlled Trials (RCTs), Case Series (CS), Retrospective Case Series (RCS) and Prospective Cohort Studies (PCS)] | Yes | American Academy of Orthopaedic Surgeons (AAOS) | No | No |
| Spinal cord surgery | Daniel et al. | 2018 | Systematic review and meta-analysis | Evaluation of IONM in the prevention of spinal cord injuries | MEDLINE (PubMed), Embase, Lilacs, and Cochrane Central Register databases of randomized assays (January 2007 to September 2017) | 2016-2006 | Six comparative clinical studies | Yes | MINORS | Yes | Random effects model |
| Carino-encephalic surgery, spine surgery, peripheral vascular surgery | Maza-Krzeptowsky et al | 2018 | Systematic review | Providing some recommendations based on the best available evidence, with the aim of standardizing methods in order to support IONM in reducing the risk of secondary nerve damage in patients undergoing carino-encephalic surgery, spine surgery, peripheral vascular surgery | American University of Beirut Medical Center (one document), PubMed Central (four documents), Springer Link (eight documents), OVIDSP (seven documents), Researchgate (two documents), Science Direct (11 documents) and Wiley Online Library (three documents) and Scottish Intercollegiate Guidelines Network (SIGN) systems in order to establish the level of evidence (LoE) and grade of recommendation (GoR). | 2017-2009 | One guideline, two systematic reviews, eight randomized clinical trial studies, 44 observational studies, and one unclassified study. | Yes | the CEPD and SIGN scales | No | - |
| Surgery of intramedullary spinal tumors | Rijs et al. | 2019 | Systematic review and meta-analysis | A summary and review of reported evidence on the use of IONM of spinal cord tumors | Embase, Medline Epub, Cochrane Central, Web of Science, and Google Scholar from January 2000 to February 2018 | 2018-2005 | Thirty one studies in qualitative analysis, 15  Meta-analysis studies | Yes | QUADAS II | Yes | Bivariate model |
| Anterior cervical procedures for spondylotic myelopathy | Thirumala et al. | 2016 | Meta-analysis | Evaluation of different IONM techniques in anterior cervical procedures | MEDLINE and the Web of Science for studies published up to February 2013 | 2012-1994 | Twenty two randomized, cohort and observational studies | No |  | Yes |  |
| Corrective surgeries for idiopathic scoliosis patients | Thirumala et al. | 2017 | Meta-analysis | Evaluation of common warning criteria and diagnostic value of MEP changes in spinal cord surgery | MEDLINE/PubMed database to determine eligible studies published before October 2014 | 2012-1998 | Twelve retrospective/prospective cohort studies | Yes | QUADAS-2 | Yes | Bivariate model |
| Spinal cord surgery | Thirumala et al. | 2017 | Meta-analysis | Evaluation of efficacy (TcMEP) in predicting impending nerve amputation during corrective spine surgery for patients with idiopathic scoliosis (IS) | PubMed/MEDLINE, Web of Science, and EMBASE from 1945 to January 2014 | 2014-1996 | Twenty five retrospective and prospective cohort studies | Yes | QUADAS-2 | Yes | Univariate random effects comparison model |
| Deformity surgery for idiopathic scoliosis | Thirumala et al. | 2016 | Meta-analysis | Determining the sensitivity, specificity, diagnostic odds ratio, and area under the receiver operating characteristic curve (ROC) curve of intraoperative SSEP/TcMEP combinations in relation to neurological outcome in patients undergoing idiopathic scoliosis correction surgery. | Pubmed/MEDLINE, Web of Science, and Embase electronic databases from January 1974 through January 2015 | 2015-2007 | 7 | Yes | QUADAS-2 | Yes | Univariate random effects |
| Deformity surgery for idiopathic scoliosis | Thirumala et al. | 2016 | Meta-analysis | Determination of diagnostic accuracy (SSEP) to predict postoperative neurological outcome in patients undergoing spinal deformity surgery for correction of adolescent idiopathic scoliosis (AIS). | MEDLINE and World Science databases from January 1950 through January 2014 | 2014-1983 | Fifteen retrospective and prospective cohort studies | Yes | QUADAS 2 | Yes | Bivariate model |
| Cervical surgery | Ajiboye et al. | 2017 | Systematic review and meta-analysis | Evaluation of sensitivity and specificity of neuromonitoring and risk of neurological damage after anterior cervical spine surgery (ACSS) with and without (ION) | Medline, Embase, Cochrane Reviews, SCOPUS, Web-of-Science (Searching Date Not Mentioned) | 2014-1996 | Nine retrospective studies and one prospective study including 26,357 patients | Yes | MINORS | Yes | Comparison of random effects/mixed effect logistic regression to compare the sensitivity and specificity of non-modal and multimodal IONM |
| Spinal cord surgery | Liu et al. | 2017 | Systematic review | Validation review of recommended warning criteria for IONM | MEDLINE, Excerpta Medica dataBASE (EMBASE),Cochrane Controlled Trials Registry, Google ScholarDatabase of Abstracts of Reviews of Effects (DARE)and Cumulative Index to Nursing and Allied HealthLiterature (CINAHL) databases after 1980. |  | Fifty two cohort study | No | -- |  | - |
| Spinal nerve surgery and monitoring | McGarvey et al. | 2014 | Systematic review | .  Investigating the evidence that IONM in the subspinal nerve affects the prevalence of post-operative shoulder diseases and predicting functional outcomes. | Medline, Scopus and Cochrane databases | 2012-1995 | Three articles | Yes | - | No | _ |
| Intramedullary spinal tumor surgery | Azad et al. | 2018 | Systematic review and meta-analysis | Evaluation of the diagnostic value of IONM in identifying postoperative injuries in IMSCT | PubMed, MEDLINE December 31, 2015 and June 30, 2016) | 2016-1993 | Fifteen retrospective cohort studies and Four prospective cohort studies and two case-control studies | Yes | American Academy of Neurology Evidence Classification System | Yes | Bivariate model and random effects |
| surgery for cervical spondylotic myelopathy | Devlin et al. | 2006 | Systematic review | Reviewing the evidence of the use of norm-monitoring technology as a diagnostic tool to evaluate nerve function during surgery for cervical spondylotic myelopathy. | National Library of Medicine (from 1996 through 2005) |  | Not reported | No | No | No | No |
| Lumbar, cervical, and thoracic surgery | Fehlings et al. | 2010 | Systematic review | Determining the sensitivity and characteristics of neuromonitoring to identify neurological injuries during spine surgery and evaluating the ability of this technique to improve clinical outcomes for patients during procedures. | MEDLINE, EMBASE, and Cochrane Collaborative Library (between 1990 and March 2009) | 2009-1986 | Thirty two retrospective and prospective cohort studies | Yes | Grading of Recommendations Assessment, Development, and Evaluation (GRADE) criteria | No | No |
