# Supplementary material for: Safety and Efficacy of Intraoperative Neuromonitoring: An Umbrella Review
Source: Health Sci Rep. 2025 Oct 13;8(10):e71370. doi: 10.1002/hsr2.71370 (PMC12516239; doi:10.1002/hsr2.71370)
Supplement: Supplementary file 7 — appendix file 7. [file HSR2-8-e71370-s003.docx]

| **Appendix 7 - Summary of the findings of studies related to the use of IONM in the surgery of spinal tumors** | | | | | | | | | |
| --- | --- | --- | --- | --- | --- | --- | --- | --- | --- |
| Author(s) | Publication Year | Included studies for meta-analysis | Overall pooled sample size | INOM modalities | Outcome measurement | Number and Type of studies included | **Sensitivity** | **Specifity** | **Other measures** |
| Ishida | 2019 | 5 | 103 | SSEPs, MEPs, NMJB, free-running EMG, and EEG | Sensitivity specificity Positive Predictive Value Negative Predictive Value | 4+1 single center cohort | IONM overall pooled diagnostic value : 77.9% (95%  CI 62.1%–88.3%), | IONM overall pooled diagnostic value :91.1% (95% CI 82.2%– 95.8%), | Positive Predictive Value: 56.7% (95% CI 27.7%–81.7%), Negative Predictive Value: 95.7% (95% CI 88.5%–98.5%) |
| Rijs et al. | 2019 | 15 | 37 | IONM (SEP, MEP, and mIONM. SEP) | Sensitivity specificity negative Likelihood Ratio Positive Likelihood Ratio Diagnostic Odds Ratio | Thirty one in qualities analysis,  15 studies in quantitative analysis | MEPs  0.838 [95% CI, 0.703-0.919 ] SSEP 0.808 [95% CI, 0.679-0.893] MIONM 0.835 [95% CI, 0.695-0.919 ] | MEPs  0.829 [95% CI, 0.536-0.843 ] SSEP 0.714 [95% CI, 0.668-0.921 ] MIONM 0579 [95% CI, 0.441-0.736 ] | **Positive Likelihood Ratio** MEPs : 4.901 SSEP:2.825 MIONM:2.072  **negative Likelihood Ratio** MEPs : 0.195 SSEP:0.269 MIONM:0.276 **Diagnostic Odds Ratio** MEPs : 29.717 SSEP:12.077 MIONM:7.507 |
| Azad et al. | 2018 | 17 | 806 | MEPs, SSEPs, dorsal column mapping, D-waves, EMG | Sensitivity pooled sensitivity specificity pooled specificity pooled DOR | Twenty one qualitative analysis studies/ 17 meta-analysis studies/15 retrospective cohort studies, and four prospective cohort studies and two case-control studies | MEPs  indididual studies range: 75% to 99%, pooled: 90% [95% CI, 84%–94%] SSEP indididual studies range:70% to 95% Pooled : of 85% (95% CI, 75–91) | MEPs indididual studies range: 27% to 97% pooled: 82% (95% CI, 70%–90%) SSEP indididual studies range:61% to 96% Pooled : 61% to 96% | Pooled DOR MEPS 55.7 (95% CI, 26.3–119 Pooled DOR SSEP 14.3 (95% CI, 5.47–37.3) MEPS pooled area under the hsROC curve  MEP:91.8%  SSEP: 86.3% |

| **Appendix 7 - Summary of the findings of studies related to the use of IONM in the surgery of spinal tumors** | | | | | | | | |
| --- | --- | --- | --- | --- | --- | --- | --- | --- |
| Author(s) | Included studies for meta-analysis | Overall pooled sample size | INOM modalities | Outcome measurement | Number and Type of studies included | Sensitivity | **Specificity** | **Other measures** |
| Thirumala et al.  2017 | 12 | 2,102 patients with idiopathic scoliosis | TcMEP | Incidence of neurological deficits, sensitivity, specificity | Prospective, or retrospective cohort reviews | 91% [95% CI 34%–100% | 96% [95% CI 92–98% | Diagnostic odds ratio:250 [95% CI 11–5767] AUV: 0.98. |
| Thirumala et al.  2016 | 7 | 2,052 patients with idiopathic scoliosis | SSEP and TcMEP | Sensitivity, specificity, and DOR |  | Pooled:  82.6% (95% CI: 56.7% - 94.5%), | Pooled: 94.4% (95% CI: 85.1% - 98.0%), | DOR:  106.16 (95% CI: 24.952 – 451.667),   AUC: 0.928,  diagnostic odds ratio |
| Thirumala et al.  2016 | 15 | 4,763 procedures on idiopathic patients | SSEP | Sensitivity and specificity of somatosensory evoked potentials to predict neurological deficits | Prospective, or retrospective cohort reviews | 84%, [95% CI 59–95%] | Pooled:98%, [95%CI 97–99%] | Diagnostic odds ratio: 340 (95% Cl 125–926) AUC: .0.99 |
| Holdefer, R. N.  2020 | 21 | 5,055 spine deformity surgeries. | MEPs, or MEPs and SEPs | Probability of a MEP deterioration which recovered by the end of surgery, P(RSC), and the conditional probability of no new post-operative deﬁcit given an RSC, P(NND\|RSC),  stratiﬁed by category of intraoperative adverse event associated with the MEP deterioration | Cohort | - | - | Probability of no new motor deﬁcit, P(NND):(r = 0.71, p < 0.001) P(RSC) for an alert associated with correction: 0.76 for osteotomies (0.48, p = 0.0008) for hypotension (0.92, p = 0.06) P(NND\|RSC) for correction: 0.94  for positioning :(0.82) for osteotomies: (0.86) for hypotension :(1.0) odds predictor of no new motor deﬁcits 25.2, p < 0.001 |

| **Appendix 7 - Summary of the findings of studies related to the use of IONM in the surgery of spinal tumors** | | | | | | | | |
| --- | --- | --- | --- | --- | --- | --- | --- | --- |
| **Author(s)** | **Included studies for meta-analysis** | **Overall pooled sample size** | **INOM modalities** | **Outcome measurement** | **Number and Type of studies included** | **Sensitivity** | **Specify** | **Other measures** |
| Ajiboye et al.  2017 | 10 | 26,357 | Multimodal IONM | Sensitivity specify overall weighted risk of neurological injury  morbidity and | Nine Retrospective and 1 Prospective | Pooled for ACSS: 71% (CI: 48%–87%) unimodal: 68% (0.39–0.88) multimodal:88% (0.04–1.0) | Pooled for ACSS: 98% (CI: 92%–100%) unimodal: 99% (CI:97%–100%) multimodal: 92% (CI: 81%–96%) | Overall weighted risk of neurological injury after ACSS: 0.64% (95% CI: 0.23–1.25) weighted risk of neurological injury for ACDFs: 0.20% (0.05–0.47)  for corpectomies: 1.02% (0.10–2.88) odds ratio, 0.726;( CI, 0.287–1.833; P=0.498). |
| Devlin et al. 2016 | - | - | (SSEPs), (tceMEPs), (EMGs) | Mortality/ reduction in neurologic deficit/ diagnoses/  Potential risk factors for electrophysiologic and  neurologic deterioration | Randomized, cohort or observational studies | Neurophysiologic monitoring is a diagnostic tool for assessment of neurologic function during cervical spine surgery. Recording of somatosensory evoked potentials (SSEPs), transcranial electrical motor evoked potentials (tceMEPs), and electromyograms (EMGs) may be useful as these monitoring modalities provide complementary information. | | |
| Fehlings et al 2010 | - |  |  | Sensitivity, specificity, positive predictive value  (PPV), negative predictive value (NPV)/ perioperative neurologic injury (spinal cord injury) / reduce perioperative neurologic complications after spine surgery. | Thirty two (Retrospective and cohort prospective) | . Based on strong evidence that multimodality intraoperative neuromonitoring (MIOM) is sensitive and specific for detecting intraoperative neurologic injury during spine surgery, it is recommended that the use of MIOM be considered in spine surgery where the spinal cord or nerve roots are deemed to be at risk, including procedures involving deformity correction and procedures that require the placement of instrumentation.  There is a need to develop evidence-based protocols to deal with intraoperative changes in MIOM and to validate these prospectively | | |
| Di Martino et al, 2019 | - |  | IONM and MIOM | Postoperative neurological deficits / specificity and sensitivity | [Randomized 8controlled trials (RCTs), case series (CS), retrospective case series (RCS), and prospective cohort studies (PCS)] | SSEP: 22 and 100%  MEP: between 78 and 100%  specificity ranging from 83.2 to 100%. | SSEP 100%  MEP: ranging from 83.2 to 100%. | The neurological complication rate: 2.17%, |
| Thirumala et al. 2017 | 25 | 9,409 | TcMEPs | Sensitivity and specificity of all TcMEP changes | Prospective studies, or retrospective cohort studies | 82.1% (95% confidence interval (CI): 73–88.6%; P< 0.001) Pooled estimates for ≥50% amplitude loss: 63.2% (95% CI: 47–76.8%; P = 0.108)  Pooled estimates fo>80% 71.7% (95% CI: 42–89.9%; P = 0.146) s. | 95.7% (95% CI: 93.7–97.1%, P< 0.001),  Pooled estimates for ≥50% amplitude loss 96.7% (95% CI: 95.1–97.8%; P< 0.001),  Pooled estimates fo>80% 98.3% (95% CI: 96.4–99.2%; | Pooled diagnostic odds ratio (DOR): 139.730 (95% CI: 73.579–265.354),  positive likelihood ratio (PLR) 19.093  negative likelihood ratio (NLR) 0.187 DOR for ≥50% amplitude loss: 61.027 (95% CI: 27.983 – 133.092) DOR≥80% amplitude loss was: 194.021 (95% CI: 55.688–675.987), |
